# Therapeutic role of mesenchymal stem cell‐derived extracellular vesicles in neuroinflammation and cognitive dysfunctions induced by binge‐like ethanol treatment in adolescent mice

**DOI:** 10.1111/cns.14326

**Published:** 2023-06-28

**Authors:** Susana Mellado, Carlos M. Cuesta, Sandra Montagud, Marta Rodríguez‐Arias, Victoria Moreno‐Manzano, Consuelo Guerri, María Pascual

**Affiliations:** ^1^ Department of Physiology, School of Medicine and Dentistry University of Valencia Valencia Spain; ^2^ Department of Psychobiology, Facultad de Psicología Universitat de Valencia Valencia Spain; ^3^ Neuronal and Tissue Regeneration Laboratory Príncipe Felipe Research Center Valencia Spain; ^4^ Príncipe Felipe Research Center Valencia Spain

**Keywords:** adolescence, binge‐like ethanol treatment, cognitive dysfunction, extracellular vesicles, mesenchymal stem cells, neuroinflammation

## Abstract

**Background:**

Extracellular vesicles (EVs) are heterogeneous membrane vesicles secreted by cells in extracellular spaces that play an important role in intercellular communication under both normal and pathological conditions. Mesenchymal stem cells (MSC) are anti‐inflammatory and immunoregulatory cells capable of secreting EVs, which are considered promising molecules for treating immune, inflammatory, and degenerative diseases. Our previous studies demonstrate that, by activating innate immune receptors TLR4 (Toll‐like receptor 4), binge‐like ethanol exposure in adolescence causes neuroinflammation and neural damage.

**Aims:**

To evaluate whether the intravenous administration of MSC‐derived EVs is capable of reducing neuroinflammation, myelin and synaptic alterations, and the cognitive dysfunction induced by binge‐like ethanol treatment in adolescent mice.

**Materials & Methods:**

MSC–derived EVs obtained from adipose tissue were administered in the tail vein (50 microg/dose, one weekly dose) to female WT adolescent mice treated intermittently with ethanol (3.0 g/kg) during two weeks.

**Results:**

MSC‐derived EVs from adipose tissue ameliorate ethanol‐induced up‐regulation of inflammatory genes (e.g., COX‐2, iNOS, MIP‐1α, NF‐κB, CX3CL1, and MCP‐1) in the prefrontal cortex of adolescent mice. Notably, MSC‐derived EVs also restore the myelin and synaptic derangements, and the memory and learning impairments, induced by ethanol treatment. Using cortical astroglial cells in culture, our results further confirm that MSC‐derived EVs decrease inflammatory genes in ethanol‐treated astroglial cells. This, in turn, confirms in vivo findings.

**Conclusion:**

Taken together, these results provide the first evidence for the therapeutic potential of the MSC‐derived EVs in the neuroimmune response and cognitive dysfunction induced by binge alcohol drinking in adolescence.

## Graphical abstract

Schematic representation of the protective effects of MSC‐EVs administration on ethanol‐induced neuroinflammation. MSC‐EVs ameliorates ethanol‐induced up‐regulation in inflammatory genes in the prefrontal cortex of adolescent mice. Notably, MSC‐EVs treatment also restored the myelin and synaptic derangements, as well as memory and learning impairments, induced by ethanol administration.

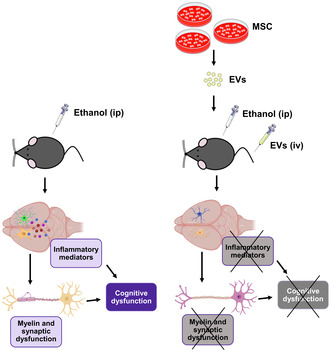

## 1 INTRODUCTION

Mesenchymal stem cells (MSC) are adult stem cells capable of stimulating the maintenance, growth, and survival of other cells.^1^ Their therapeutic potential seems to be mediated by the paracrine factors contained in microvesicles.^2^ Indeed the extracellular vesicles (EVs) that derive from MSC (MSC‐EVs) display a different genetic cargo and protein content, which play a significant role in biological processes, including regulation of inflammation, apoptosis, angiogenesis, adipogenesis, blood coagulation, and extracellular matrix remodeling.^3^ However, MSC‐EVs have emerged as therapeutic agents to reduce tissue injury and enhance tissue repair in cerebral injury, lung injury, myocardial injury, and kidney injury cases.^4^, ^5^

Extracellular vesicles are microvesicles that originate from multivesicular bodies and can be isolated from diverse body fluids and multiple cell cultures supernatants.^6^ The content of EVs is complex in nature and includes several types of proteins, RNAs, miRNAs, enzymes, and lipids, which act as messengers during cell‐to‐cell communication in multiple physiological and pathological processes.^7^, ^8^, ^9^ Analyses of MSC‐EVs have demonstrated that their content is enriched in miRNAs and proteins.^3^ In the central nervous system, neuron‐ and astrocyte‐derived EVs play critical roles in several processes, such as promoting neurite outgrowth, synaptic plasticity, neuronal survival, and neuroprotection,^7^ MSC‐EVs can recover the neuronal and astroglial damage induced by several injuries.^10^

The adolescent brain shows important changes in its structure and function,^11^ including synaptic plasticity and neural connectivity, which especially occur in the prefrontal cortex (PFC) and other subcortical areas.^12^, ^13^ These extensive developing changes in brain maturation might explain the adolescent brain's vulnerability to the deleterious effects of ethanol.^14^, ^15^ Our previous studies have demonstrated that by activating innate immune receptors TLR4 (Toll‐like receptor 4) in glial cells, binge‐like ethanol exposure in adolescence leads to the release of cytokines and chemokines, and causes neuroinflammation and neural damage.^16^, ^17^ Activation of the TLR4 response has also been demonstrated in myelin and synaptic alterations, as well as cognitive and behavioral impairments, induced by binge drinking in adolescent mice.^18^ This scenario suggests the involvement of the neuroinflammatory immune response in behavioral deficits. Indeed human studies have shown that the one brain region most affected by ethanol drinking in adolescence is the PFC, a region associated with cognitive control and executive function.^19^ Likewise, myelin dysfunctions have been related to attention and spatial working memory deficits in human adolescents with heavy alcohol abuse.^20^

In agreement with the therapeutic beneficial effects of MSC‐EVs to treat neurological and neurodegenerative diseases,^21^ the present study provides evidence that the intravenous administration of MSC‐EVs from adipose tissue ameliorates neuroinflammation, as well as myelin and synaptic alterations, which lead to the cognitive impairments induced by binge‐like ethanol treatment in adolescent mice.

## 2 MATERIALS AND METHODS

### 2.1 MSC isolation, culture, and isolation of MSC‐EVs

Human adipose tissue was obtained from surplus fat tissue during knee prosthesis operation performed on four patients under sterile conditions. Human samples were anonymized. The experimental procedure was previously evaluated and accepted by the Regional Ethics Committee for Clinical Research with Medicines and Health Products following Code of Practice 2014/01. As the exclusion criteria, no samples were collected from patients with a history of cancer or infectious diseases at the time of (viral or bacterial) surgery. All the human patients voluntarily signed an informed consent document to use the adipose samples. Cells were expanded and grown in the growth medium [GM: High glucose DMEM basal medium supplemented with 20% FBS (previously centrifuged at 100 000×*g* for 1 h for EV depletion and then filtered by a 0.2 μm filter), 100 units/mL penicillin, 100 μg/mL streptomycin and 2 mM l‐glutamine in]. MSC have been characterized and previously described.^22^, ^23^ Subconfluent cells were incubated in GM for 48 h. Then, media were collected and cleared from detached cells and cell fragments by centrifugation at 300×*g* for 10 min, and by the supernatant at 2000×*g* for 10 min, respectively. Subsequently, apoptotic bodies and other cellular debris were pelleted by centrifuging the resulting supernatant at 10 000×*g* for 30 min. EVs were then pelleted from the previous resulting supernatant at 100 000×*g* for 1 h. The EVs pellet was washed with PBS and centrifuged at 100 000×*g* for 1 h. EVs were finally suspended in 100 mL PBS and stored at −80°C.

### 2.2 Animals and treatments

Ninety‐two female C57BL/6 WT (wild‐type) mice (Harlan Ibérica) were used. Mice were housed (3–4 animals/cage) and maintained on a water and solid diet ad libitum. Environmental conditions, such as light and dark (12/12 h), temperature (23°C), and humidity (60%), were controlled for all the animals. All the animal experimental procedures were approved by the Ethical Committee of Animal Experimentation of the Principe Felipe Research Center (Valencia, Spain), following the guidelines approved by European Communities Council Directive (86/609/ECC) and Spanish Royal Decree 53/2013 modified by Spanish Royal Decree 1386/2018.

The intermittent ethanol treatment was initiated early in adolescence or during the prepubescent period on postnatal day (PND) 30.^24^ Morning doses of either saline or 25% (v/v) ethanol (3 g/kg) in isotonic saline were administered intraperitoneally to 30‐day‐old mice on two consecutive days with 2‐day gaps without injections for 2 weeks (PND30–PND43), as previously described.^18^, ^25^ No signs of peritoneal cavity irritation, pain or distress, or peripheral inflammation induced by intraperitoneal ethanol concentration were noted, which agrees with other studies that have used intraperitoneal ethanol administration.^26^ After a single ethanol dose, blood alcohol levels peaked at 30 min (~340 mg/dL) and then progressively lowered until 5 h post‐injection. Three hours before ethanol administration, animals were also treated with MSC‐EVs (50 μg/dose) or saline (sodium chloride, 0.9%) in the tail vein once a week (with the third and seventh ethanol dose). Animals were randomly assigned to four groups according to their treatments: (1) physiological saline or control; (2) physiological saline plus MSC‐EVs; (3) ethanol; (4) ethanol plus MSC‐EVs. No changes in either animals' body weight or brain weight were observed during the intermittent treatment (Figure S1). Animals were anesthetized 24 h after the last (8th) ethanol or saline administration (PND 44). Brains were removed and transferred to a plate placed on ice. Olfactory bulbs, cerebellum, and pons were removed. Brains were placed with the ventral side facing the plate. We used curved forceps to remove the cortical hemispheres from the rest of the brain. Then the PFC dissection was performed based on visual information and using brain atlas coordinates.^27^, ^28^ PFCs (*n* = 9–10 mice/group) were immediately snap‐frozen in liquid nitrogen and stored at −80°C until used. In addition, some animals were anesthetized, perfused with paraformaldehyde/glutaraldehyde, and used for the electron microscopy analysis (*n* = 6 mice/group). Other animals (*n* = 10–12 mice/group) were employed to perform behavioral studies after ethanol treatment on PND 50 in this test daily order: novel object recognition, passive avoidance, and Hebb‐Williams maze.

### 2.3 Primary culture of astrocytes and treatments

Astroglial cells (98 ± 0.5% GFAP‐positive cells)^29^ were obtained from the brain cortices of newborn WT pups (6–8 animals per culture; *n* = 6). They were mechanically dissociated and cultured in DMEM (Dulbecco's modified eagle's medium), supplemented with 20% fetal bovine serum (FBS), 100 U/mL penicillin/streptomycin, 2.5 μg/mL fungizone, 2 mM glutamine and 1 g/L glucose, and seeded at 850 cells/mm^2^. On day 7 in vitro, FBS was reduced to 10% and glucose was removed. On day 14 in vitro, cell cultures were 90%–95% confluent and FBS was replaced with bovine serum albumin (BSA, 1 mg/mL) 24 h prior to ethanol (40 mM) stimulation to avoid EVs from being present in FBS. Some plates were incubated with MSC‐EVs (2.5 μg) 2 h before the ethanol treatment. After 24 h of ethanol stimulation, cells were harvested, frozen, and stored at −80°C until further use.

### 2.4 Extracellular vesicles characterization by transmission electron microscopy and nanoparticles tracking analysis

The freshly isolated EVs were fixed with 2% paraformaldehyde and prepared as previously described.^30^ Preparations were examined under a transmission FEI Tecnai G2 Spirit electron microscope (FEI Europe) with a Morada digital camera (Olympus Soft Image Solutions GmbH). In addition, an analysis of the absolute size range and concentration of microvesicles was performed using NanoSight NS300 Malvern (NanoSight Ltd.), as previously described.^30^

### 2.5 Western blot analysis

The Western blot technique was performed in MSC‐EVs for their characterization, as described elsewhere.^18^ The employed primary antibodies were: anti‐CD9, anti‐CD63, anti‐CD81, and anti‐calnexin (Santa Cruz Biotechnology). Membranes were washed, incubated with the corresponding HRP‐conjugated secondary antibodies, and developed by the ECL system (ECL Plus; Thermo Scientific). The cell lysate from the astrocyte primary cell culture was used as the negative control for CD9, CD63, and CD81, and as the positive control for calnexin. The full unedited blots are included in the Supplementary Material.

### 2.6 RNA isolation, reverse transcription, and quantitative RT‐PCR

The frozen PFC samples (10–20 mg) and astroglial cells were used for total RNA extraction. Tissue and cells were disrupted using 1 mL of TRIzol (Sigma‐Aldrich), and the total RNA fraction was extracted following the manufacturer's instructions. Total mRNA was reverse‐transcribed by the NZY First‐Strand cDNA Synthesis Kit (NZYTech, Lda. Genes and Enzymes). Quantitative two‐step RT‐PCR (real‐time reverse transcription) was performed with the Light‐Cycler 480 detection System (Roche Diagnostics). Genes were amplified employing the AceQ® qPCR SYBR Green Master Mix (NeoBiotech) following the manufacturer's instructions. The mRNA level of housekeeping gene cyclophilin A was used as an internal control for the normalization of the analyzed genes. All the RT‐qPCR runs included non‐template controls (NTCs). Experiments were performed in triplicates. Quantification of expression (fold change) from the Cq data was calculated by the ΔΔCq method^31^ by the LightCycler 480 relative quantification software (Roche Diagnostics). Details of the nucleotide sequences of the used primers are found in the Supplementary Material (Table S1).

### 2.7 Brain tissue preparation and electron microscopy

Mice were anesthetized by an intraperitoneal injection of sodium penthobarbital (60 mg/kg) and fentanyl (0.05 mg/kg) for analgesia. Animals were then perfused transcardially with 0.9% saline containing heparin, followed immediately by 2% paraformaldehyde and 2.5% glutaraldehyde in 0.1 M phosphate buffer, pH 7.4, for tissue fixation. The fixed brains were removed, postfixed overnight at 4°C with the same fixative solution, and then stored at 4°C in PBS. After removing the olfactory bulbs, the anterior coronal section, of approximately 1 mm was discarded. The following section of approximately 1 mm from 2.5 to 1.5 mm anterior to Bregma was used to cut PFCs in sections of 200 μm on a Leica VT‐1000 vibratome (Leica). Sections were post‐fixed with 2% osmium, rinsed, dehydrated, and embedded in Durcupan resin (Fluka, Sigma‐Aldrich). Semithin sections (1.5 μm) were cut with an Ultracut UC‐6 (Leica) and stained lightly with 1% toluidine blue. Finally, ultrathin sections (0.08 μm) were cut with a diamond knife, stained with lead citrate (Reynolds solution), and examined under a transmission FEI Tecnai G2 Spirit electron microscope (FEI Europe) using a Morada digital camera (Olympus Soft Image Solutions GmbH). Images were analyzed by the ImageJ software (version 1.53a). Synaptic and myelin quantifications were carried out on 4–5 sections per PFC. For each group, 150–175 postsynaptic density thicknesses and 150–175 synaptic cleft width were analyzed. In addition, the number of synaptic vesicles was quantified in 75–100 presynaptic terminals. Damage in the total myelin sheaths was analyzed by measuring the alterations of the total multiple layers of myelin membrane around an axon and was represented as a percentage. Myelin quantification was measured in at least 40–45 axons chosen randomly from each group.

### 2.8 Behavioral testing

#### 2.8.1 Novel object recognition test

Mice performed this test in a black open box (24 cm × 24 cm × 15 cm) using small nontoxic objects: two plastic boxes and a plastic toy. The task procedure is described elsewhere^25^ and consists of three phases: habituation, training session (T1), and test session (T2). During the habituation session, mice spent 5 min exploring the open‐field arena where T1 and T2 were performed. During the training session, one mouse was placed in the open‐field arena containing two identical sample objects placed in the middle of the testing box for 3 min. After a 1‐min retention interval, the animal was returned to the open‐field arena with two objects during the test session (3 min): one object was identical to the sample and the other was novel. Object exploration was defined as the orientation of the animal's snout toward the object within a range of ≤2 cm from the object. The recognition index was calculated by measuring the discrimination index [D.I. = (tnovel – tfamiliar)/(tnovel + tfamiliar) × 100%], with “t” taken as the time that each mouse spent exploring an object.

#### 2.8.2 Passive avoidance test

Step‐through inhibitory avoidance apparatus for mice (Ugo Basile) was employed for the passive avoidance test. The cage was made of Perspex sheets and was divided into two compartments (15 × 9.5 × 16.5 cm each). The safe compartment was white and illuminated by a light fixture (10 W) fastened to the cage lid. The “shock” compartment was dark and made of black Perspex panels. Both compartments were divided by a door that automatically operated by sliding on the floor. The floor was made of 48 stainless steel bars (0.7 mm in diameter) placed 8 mm apart. Passive avoidance tests were run following the previously described procedure.^32^ On the training day, each mouse (*n* = 10–12 mice/group) was placed inside the illuminated compartment facing away from the dark compartment. The door leading to the dark compartment was opened after a 60‐s habituation period. When the animal had placed all four paws in the dark compartment, a footshock (0.5 mA, 3 s) was delivered and the animal was immediately removed from the apparatus and returned to its home cage. The time taken to enter the dark compartment (step‐through latency) was recorded. Retention was tested 24 h and 7 days later following the same procedure, but with no footshock. The maximum step‐through latency lasted 300 s.

#### 2.8.3 Hebb‐Williams maze

This task was used for the advantages that it offers over other tests because not only can problem‐solving, visuo‐spatial abilities, and cognitive performance in rodents^33^ be assessed, but so can easy and difficult learnings and, consequently, minor cognitive deficits can be differentiated. Motivation to perform this maze is not based on reinforcement (i.e., food), but on escaping from a stressful situation (cold water), which can influence learning and memory.^34^, ^35^

The maze used in our experiments was made of black plastic and measured 60 cm wide × 60 cm long × 10 cm high. It contained a start box and a goal box (both 14 cm wide × 9 cm long), which were positioned in diagonally opposite corners. The maze contained cold water at a wading depth (15°C, 3.5 cm high), while the goal box was stocked with fresh dry tissue. Several maze designs were produced by fixing different arrangements of barriers to a clear plastic ceiling. This apparatus allows the cognitive process of routed learning and water‐escape motivation to be measured. The following procedure was based on that employed by,^36^ in which mice must navigate the maze and cross over from the wet start box to the dry goal box to escape cold water. Animals (*n* = 10–12 mice/group) underwent a 5‐min habituation period (dry sand, no barriers) on day 1, and faced problem A on day 2 and problem D on day 3 (4 trials/day) (practice mazes). Mice were subsequently submitted to mazes 1, 5, 3, 4, and 8 on separate days on which eight trials took place. The time limit for reaching the goal box was 5 min, after which time the mouse was guided to the box. The following measurements were recorded: total latency score (the sum of the latencies in all the problem trials in each maze); latency to reach the goal during the eighth trial; error scores, for which a similar total was used (where “error” was considered the act of entering the error zone as previously described). Following,^37^ mazes were defined as easy (1, 3, and 4) or difficult (5 and 8).

### 2.9 Statistical analysis

The results are reported as the mean ± SEM. All the employed statistical parameters were calculated with SPSS v28. The Shapiro–Wilk test was used to test for data distribution normality. A one‐way ANOVA or a two‐way ANOVA was used as parametric tests, and the Mann–Whitney *U* test or the Kruskal–Wallis test was used as non‐parametric alternatives. Values of *p* < 0.05 were considered statistically significant.

Electron microscopy imaging was analyzed with a one‐way ANOVA. Quantitative RT‐PCR and behavioral data from the novel object recognition were analyzed by a two‐way ANOVA with two between‐subject variables: ethanol (saline and ethanol); MSC‐EVs (with and without EVs). Bonferroni adjustment was employed for the post hoc comparisons in the ANOVA. The number of errors in the Hebb‐Williams maze data was analyzed by a two‐way ANOVA with the same two between‐subject variables in the easy and difficult mazes. As normal distribution was lacking, the time to reach the goal and the particular measures of each trial in the easy and difficult mazes of the Hebb‐Williams maze, and the passive avoidance test data, were analyzed by the Kruskal‐Wallis test and pairwise comparisons by the Mann–Whitney *U* test.

## 3 RESULTS

### 3.1 MSC‐EVs ameliorate the neuroinflammatory immune response induced by binge‐like ethanol treatment in adolescent mice

**FIGURE 1:**
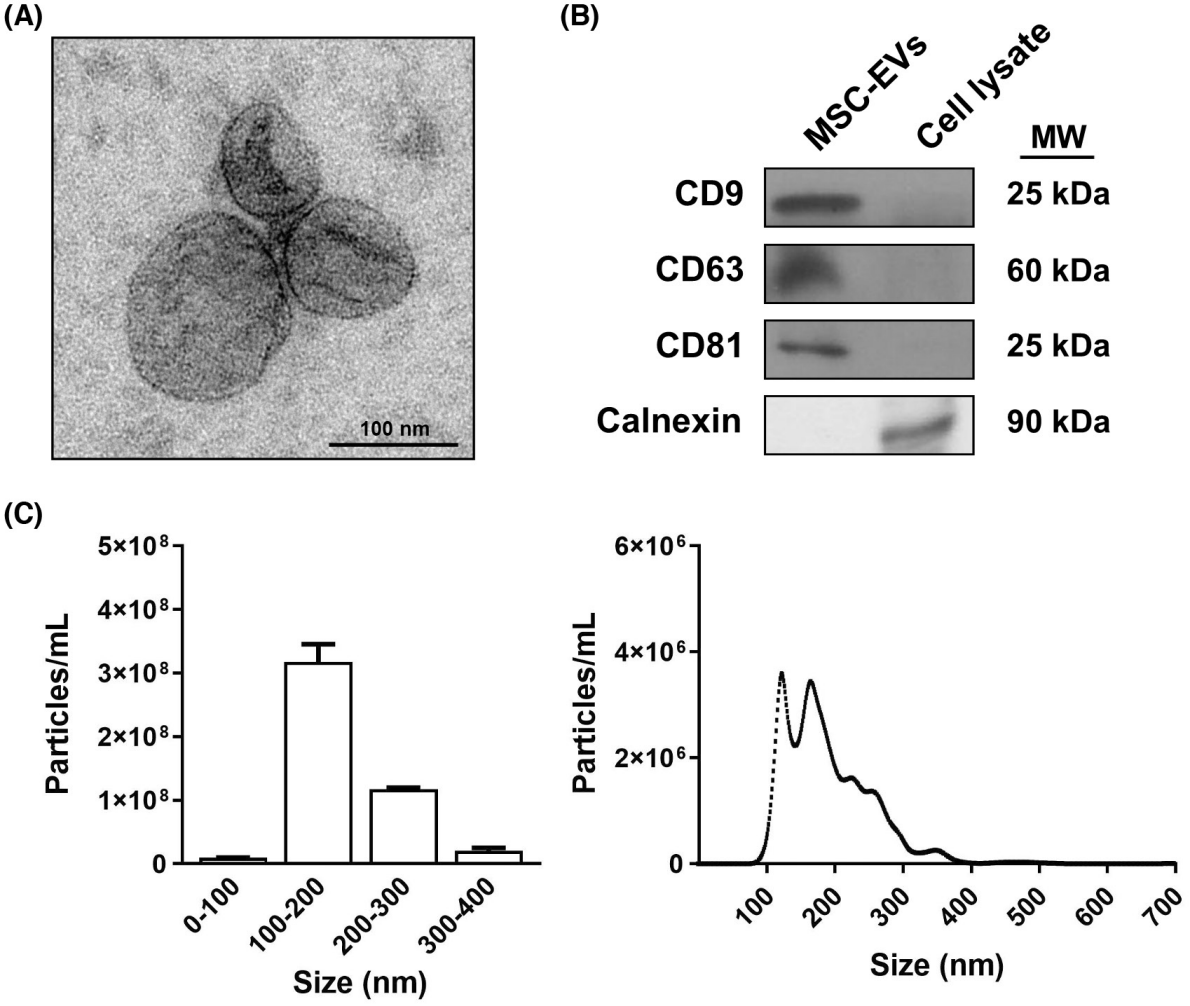
Characterization of MSC‐EVs. (A) Electron microscopy image of MSC‐EVs. (B) Analysis of the protein expression of EV markers, such as CD9, CD63, and CD81 in EVs and the cell lysate. Calnexin expression was also used to discard cytosolic protein contamination in EV samples. The cell lysate from the astrocytes in the culture was used as the positive control. A representative immunoblot for each protein is shown. (C) Measurement of the size distribution and concentration of MSC‐EVs by the nanoparticles tracking analysis.

We first characterized MSC‐EVs by electron microscopy, nanoparticle tracking analysis, and EVs markers (Figure 1). The size distribution and concentration of the MSC‐secreted nanoparticles using the NanoSight resulted in a high peak that ranged between 100 and 200 nm, which included the size range of EVs as demonstrated by electron microscopy. In addition, these EVs expressed the exosome markers, named tetraspanin proteins (CD63, CD9, and CD81), whereas no signs of cytosolic protein contamination were observed by the calnexin protein.

**FIGURE 2:**
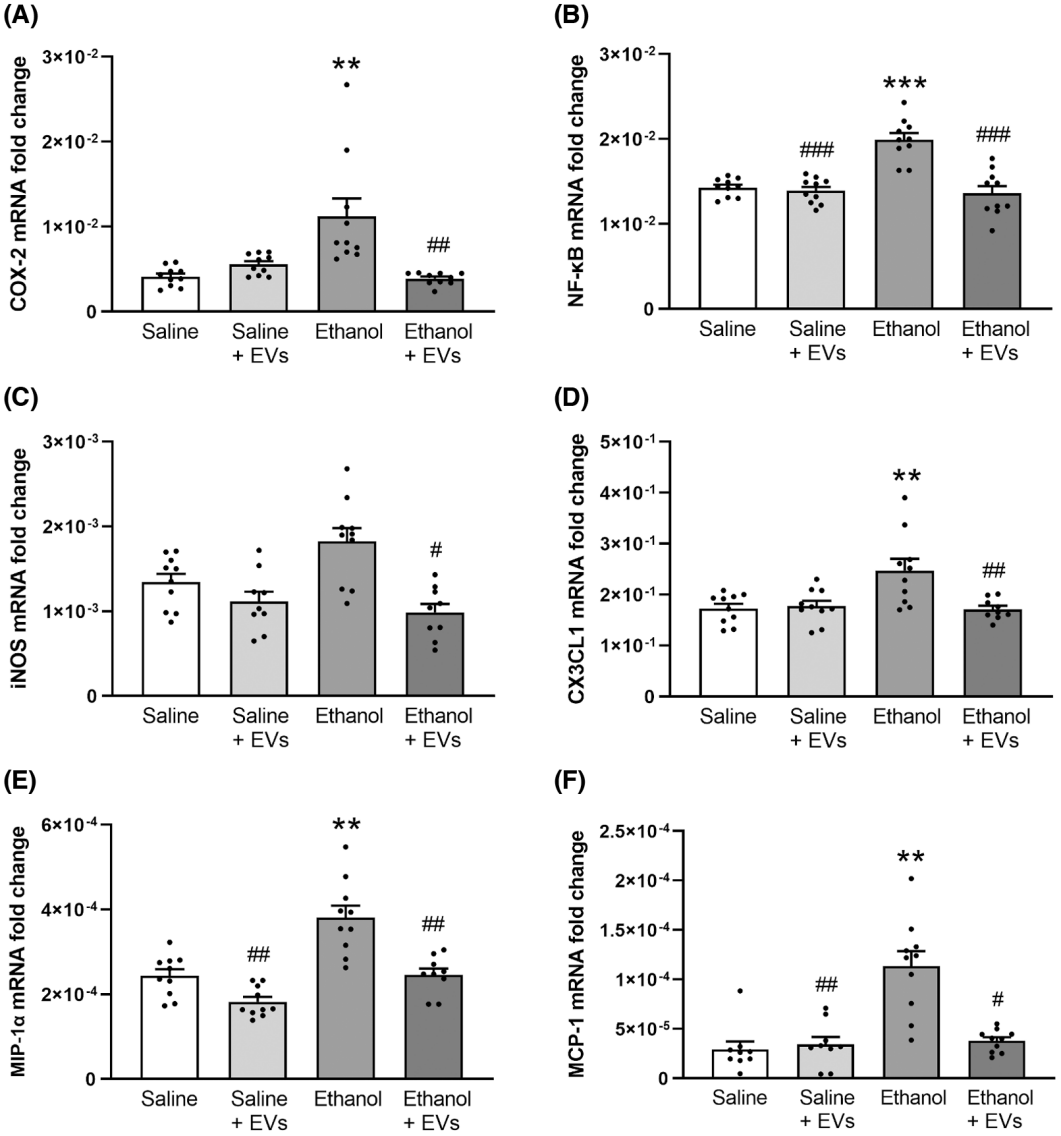
MSC‐EVs restore the levels of inflammatory genes induced by binge‐like ethanol treatment in adolescent mice. The mRNA levels of COX‐2 (A), NF‐κB (B), iNOS (C), CX3CL1 (D), MIP‐1α (E), and MCP‐1 (F) were analyzed in the PFC samples of adolescent mice. Data represent mean ± SEM, *n* = 9–10 mice/group. **p < 0.01 and ***p < 0.001, compared to their respective saline‐treated group; ^#^ *p* < 0.05, ^##^ *p* < 0.01, and ^###^ *p* < 0.001, compared to their respective ethanol‐treated group.

Then we analyzed if MSC‐EVs administration can restore the up‐regulation in the mRNA levels of inflammatory genes induced by ethanol treatment. For these experiments, we used adipose MSC‐EVs intravenously administered 3 h before ethanol injection in adolescent mice (see 2. Materials and Methods). We measured the COX‐2, NF‐κB, iNOS, CX3CL1, MIP‐1α, and MCP‐1 levels in the PFC under different experimental conditions. Figure 2 shows that ethanol treatment increased the gene expression of COX‐2 [*F*(3, 36) = 12.541, *p* < 0.01; Figure 2A], NF‐κB [*F*(3, 36) = 21.753, *p* < 0.001; Figure 2B], CX3CL1 [*F*(3, 35) = 7.262, *p* < 0.01; Figure 2D], MIP‐1α [*F*(3, 35) = 20.902, *p* < 0.01; Figure 2E] and MCP‐1 [*F*(3, 34) = 17.718, *p* < 0.01; Figure 2F] compared to their saline counterparts. Notably, MSC‐EVs administration was able to attenuate the ethanol‐induced expression of these proinflammatory molecules compared to the ethanol‐treated mice COX‐2 [*F*(3, 36) = 12.541, *p* < 0.01; Figure 2A], NF‐κB [*F*(3, 36) = 21.753, *p* < 0.001; Figure 2B], CX3CL1 [*F*(3, 35) = 7.262, *p* < 0.01; Figure 2D], MIP‐1α [*F*(3, 35) = 20.902, *p* < 0.01; Figure 2E] and MCP‐1 [*F*(3, 34) = 17.718, *p* < 0.05; Figure 2F]. However, iNOS expression (Figure 2C) showed a tendency to increase in the ethanol‐treated mice. This expression significantly decreased in the animals treated with ethanol plus MSC‐EVs compared to the ethanol‐treated mice [*F*(3, 34) = 7.541, *p* < 0.05]. In addition, ethanol treatment significantly increased the NF‐κB [*F*(3, 36) = 21.753, *p* < 0.001; Figure 2B], MIP‐1α [*F*(3, 35) = 20.902, *p* < 0.01; Figure 2E] and MCP‐1 [*F*(3, 34) = 17.718, *p* < 0.01; Figure 2F] levels compared to the saline plus MSC‐EVs‐treated mice. No changes in the expression of these genes were found between the saline‐ and MSC‐EVs‐treated animals.

### 3.2 MSC‐EVs reduce the myelin and synaptic alterations in the PFC of ethanol‐treated adolescent mice

**FIGURE 3:**
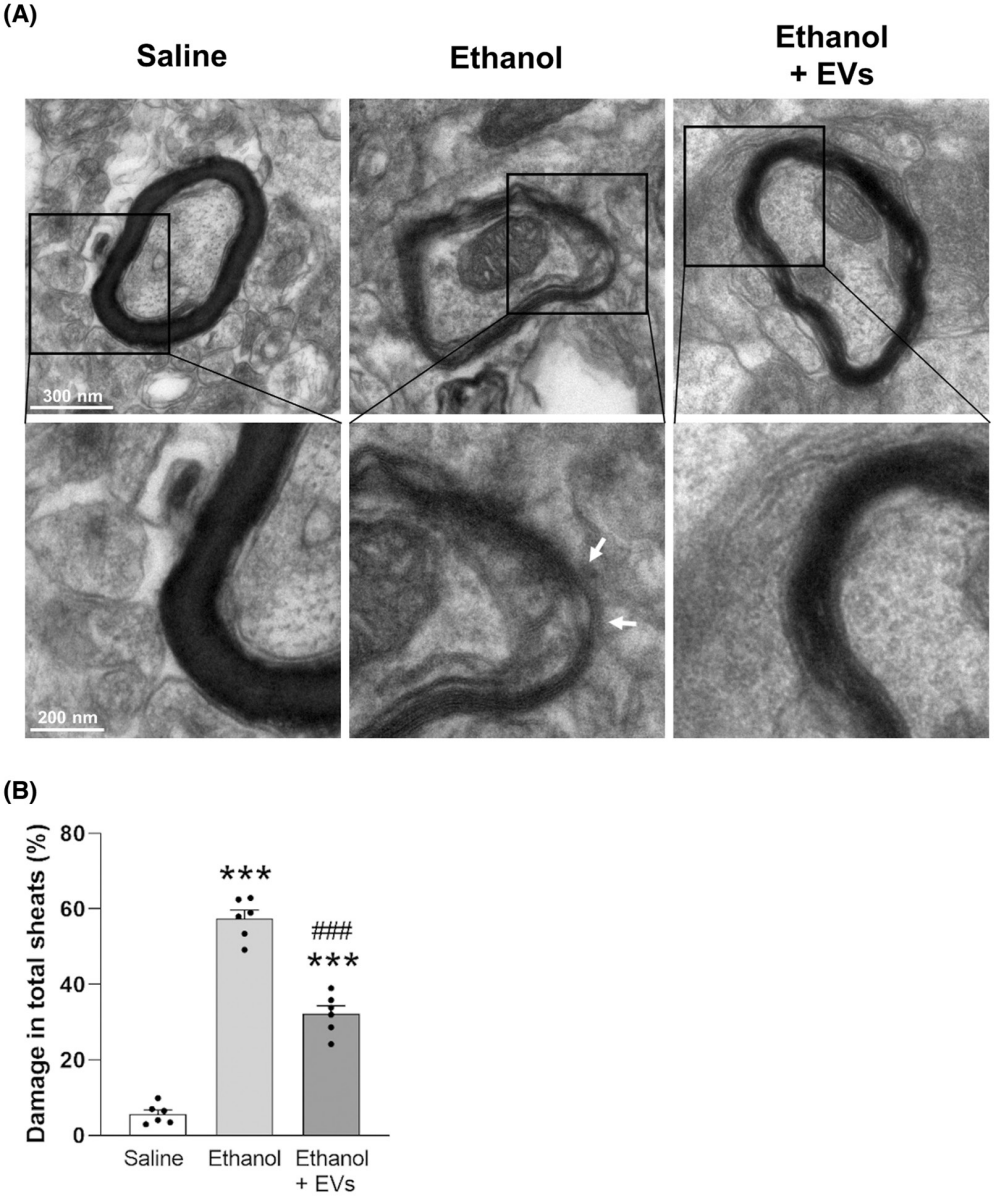
MSC‐EVs diminish the myelin sheath disarrangements induced by binge‐like ethanol treatment in adolescent mice. (A) The representative transmission electron micrographs of the PFC of the adolescent mice treated with saline, ethanol, and ethanol plus EVs are shown. Arrows indicate the interlaminar splitting of myelin sheaths. (B) Bars represent the percentage of myelin sheath damage. Data denote mean ± SEM, *n* = 6 mice/group. ***p < 0.001, compared to the saline‐treated group; ^###^ *p* < 0.001, compared to the ethanol‐treated group.

**FIGURE 4:**
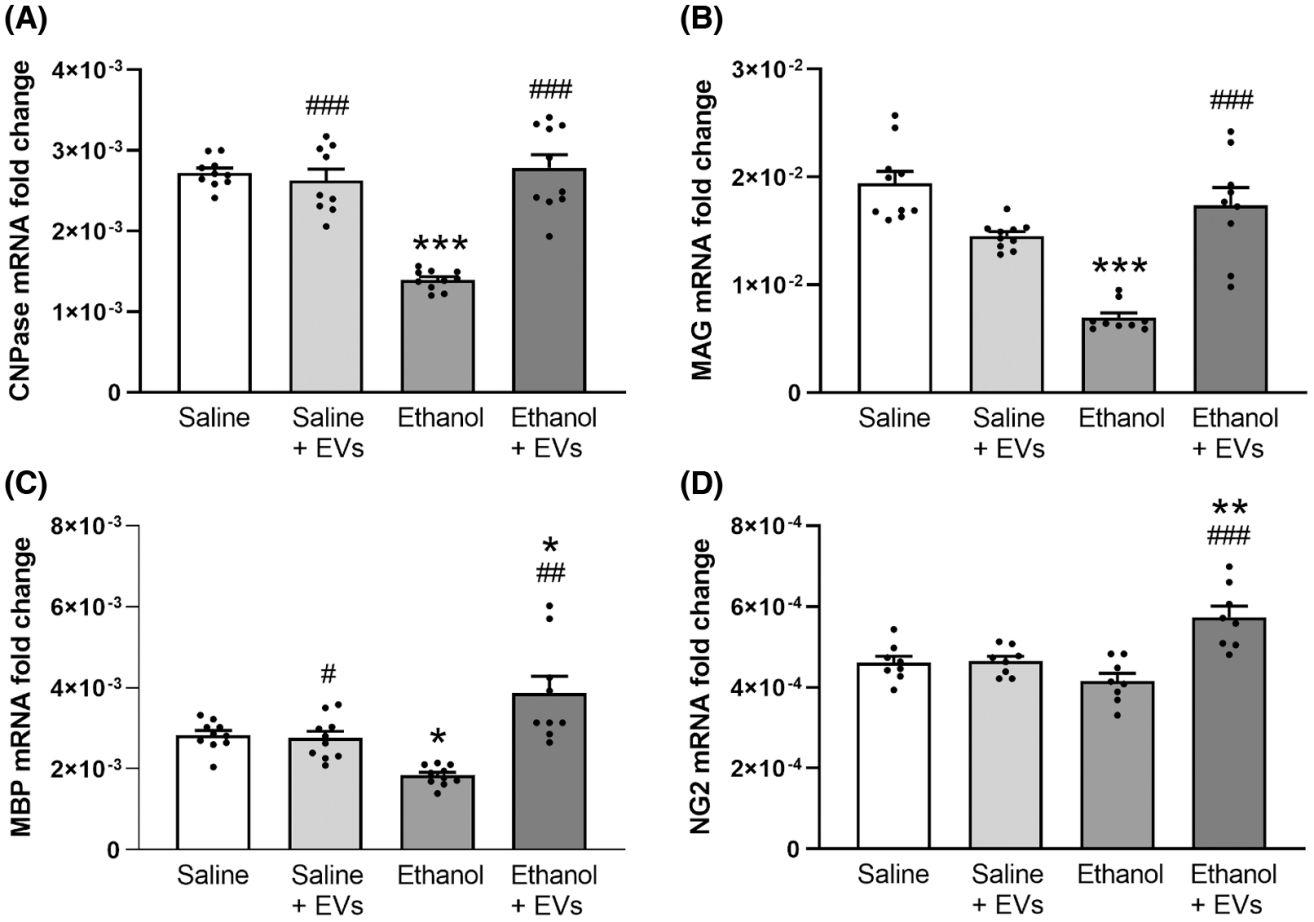
MSC‐EVs restore the levels of the myelin‐related genes induced by binge‐like ethanol treatment in adolescent mice. The mRNA levels of CNPase (A), MAG (B), MBP (C), and NG2 (D) were analyzed in the PFC samples of adolescent mice. Data represent mean ± SEM, *n* = 9–10 mice/group. *p < 0.05, **p < 0.01 and ***p < 0.001, compared to their respective saline‐treated group; ^#^ *p* < 0.05, ^##^ *p* < 0.01 and ^###^ *p* < 0.001, compared to their respective ethanol‐treated group.

Considering that intermittent binge‐like ethanol drinking alters the myelin structure in the PFC of adolescent mice,^18^ we herein analyzed the potential beneficial role of MSC‐EVs in ethanol‐induced myelin structure alterations. Figure 3A shows that while ethanol treatment induces the irregular shapes of myelin fibers and causes interlaminar splitting of myelin sheaths (Figure 3A, arrows), the administration of MSC‐EVs ameliorates the ethanol effects. Likewise, the analysis of damage in the total myelin sheaths increased in the animals treated with ethanol [*F*(2, 15) = 190.940, *p* < 0.001], whereas the MSC‐EVs injection was able to restore this alteration in ethanol‐treated mice [*F*(2, 15) = 190.940, *p* < 0.001; Figure 3B]. However, significant differences in the total myelin sheaths were observed between the animals treated with saline and ethanol plus MSC‐EVs [*F*(2, 15) = 190.940, *p* < 0.001]. We next determined the expression of several myelin‐related genes in the PFC of the adolescent mice treated with ethanol and/or MSC‐EVs. Figure 4 shows that EVs administration was able to restore the decrease in the gene expression of CNPase [*F*(3, 35) = 40.804, *p* < 0.001; Figure 4A], MAG [*F*(3, 34) = 28.772, *p* < 0.001; Figure 4B] and MBP [*F*(3, 35) = 17.747, *p* < 0.01; Figure 4C] induced by ethanol in adolescent mice. Nonetheless, NG2 expression (Figure 4D) showed a tendency to decrease in the ethanol‐treated mice, and its expression significantly increased in the animals treated with ethanol plus MSC‐EVs compared to the ethanol‐treated mice [*F*(3, 32) = 14.423, *p* < 0.001]. However, no changes were found in the expression of these genes between the saline‐treated and EVs‐treated animals.

**FIGURE 5:**
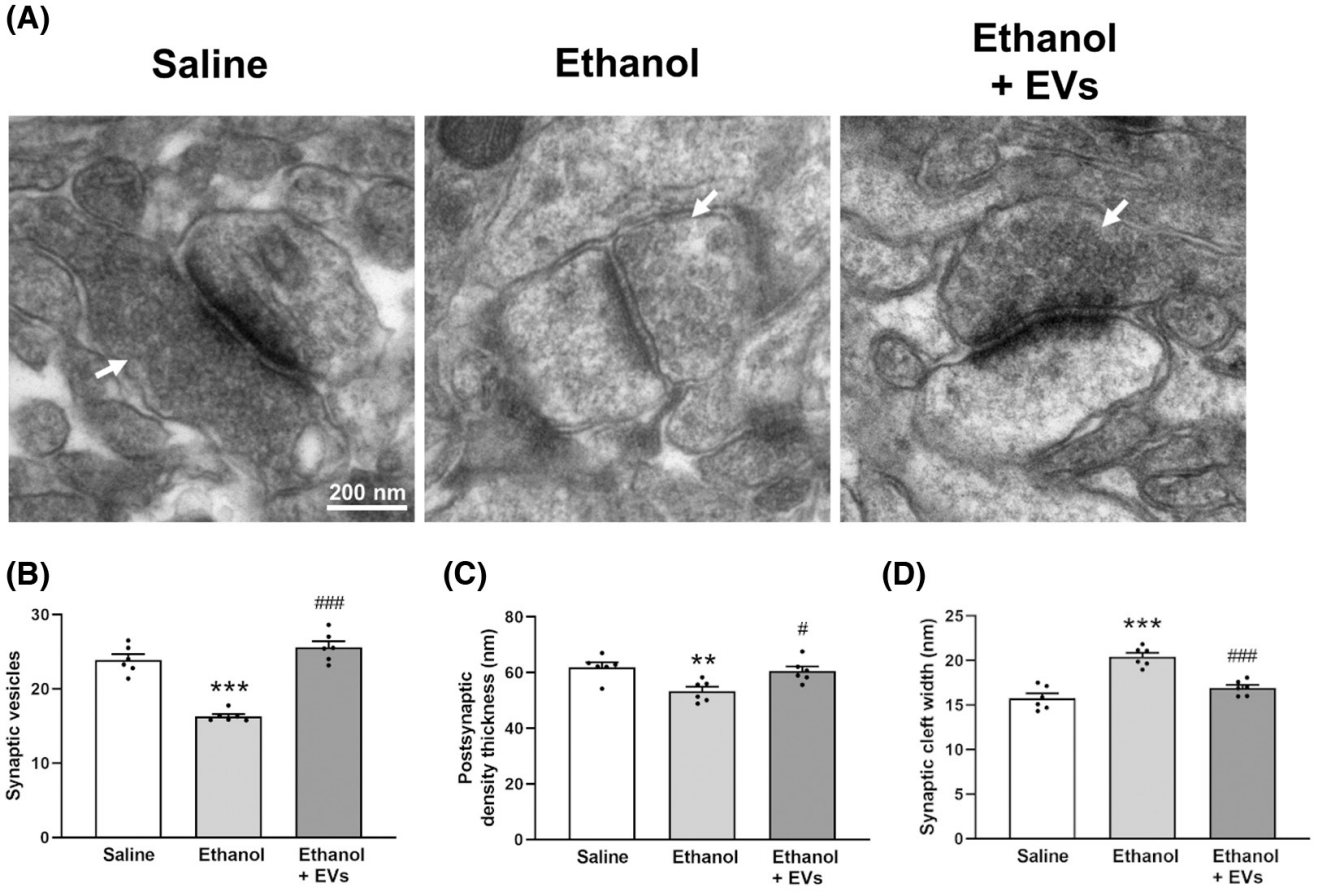
The electron microscopy analysis shows the role of MSC‐EVs in the synaptic structure induced by binge‐like ethanol treatment in adolescent mice. (A) Representative transmission electron micrographs from the PFC of the adolescent mice treated with saline, ethanol, and ethanol plus EVs. Arrows mark vesicles. Bars represent synaptic vesicle number (B), postsynaptic density thickness (C), and synaptic cleft width (D). Data represent mean ± SEM, *n* = 6 mice/group. **p < 0.01 and ***p < 0.001, compared to their respective saline‐treated group; ^#^ *p* < 0.05 and ^###^ *p* < 0.001, compared to their respective ethanol‐treated group.

We also assessed the potential role of MSC‐EVs in the ultrastructural alterations in the synaptic elements induced by intermittent ethanol treatment in adolescent mice (Figure 5A), as previously demonstrated.^18^ To accomplish this, we analyzed vesicle number, synaptic cleft width, and postsynaptic density thickness in the PFC of adolescent mice. Figure 5 shows that MSC‐EVs were able to restore: (1) decreases in the number of vesicles [*F*(2, 15) = 57.275, *p* < 0.001; Figure 5B]; (2) reduction in postsynaptic density thickness [*F*(2, 15) = 7.840, *p* < 0.05; Figure 5C]; (3) increases in synaptic cleft width [*F*(2, 15) = 29.120, *p* < 0.001; Figure 5D] in the ethanol‐treated adolescent mice number of vesicles [*F*(2, 15) = 57.280, *p* < 0.001; Figure 5B], postsynaptic density thickness [*F*(2, 15) = 7.840, *p* < 0.01; Figure 5C] and synaptic cleft width [*F*(2, 15) = 29.120, *p* < 0.001; Figure 5D]. No significant differences were noted between the animals treated with saline and ethanol plus EVs.

### 3.3 MSC‐EVs restore the cognitive dysfunctions induced by binge‐like ethanol treatment in adolescent mice

We next assessed whether the amelioration of the neuroinflammation, myelin and synaptic alterations induced by MSC‐EVs in the ethanol‐treated adolescent mice would also be capable of restoring cognitive dysfunction. We performed several memory and learning tasks, such as the passive avoidance test, the novel object recognition test, and Hebb‐Williams maze. These paradigms have been validated and used fairly often in animal cognitive research, to evaluate cognitive aspects, such as recognition ability, short‐term/working memory, short‐term and long‐term memory, and also the learning process.^18^, ^32^, ^38^, ^39^, ^40^, ^41^

**FIGURE 6:**
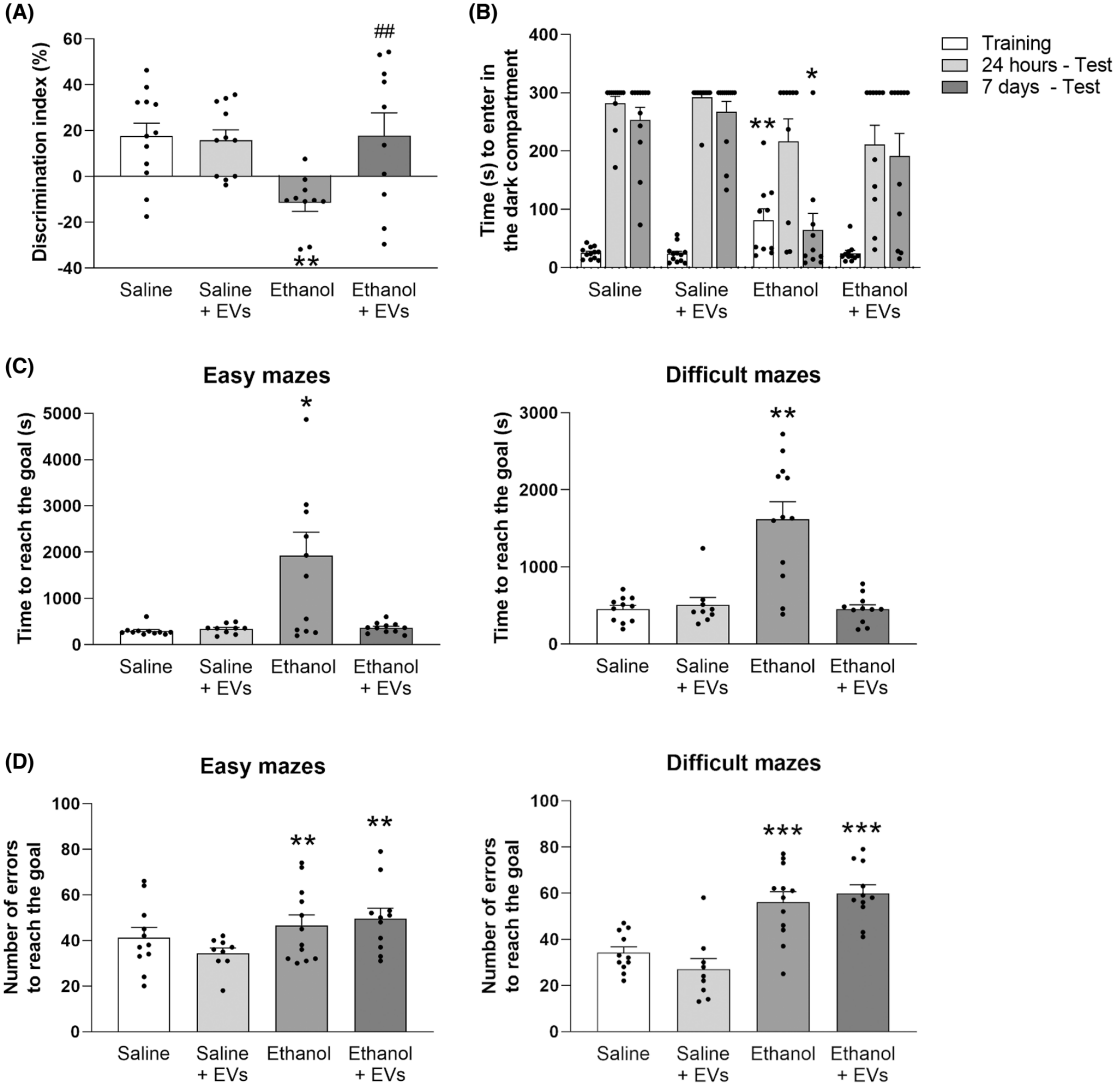
MSC‐EVs restore ethanol‐induced cognitive dysfunction in adolescent mice. (A) Bars represent the mean (±SEM, *n* = 10–12 mice/group) of the discrimination index during the novel object recognition task. **p < 0.01 compared to their respective saline‐treated group. ^##^ *p* < 0.01 compared to the ethanol‐treated group. (B) Bars represent the time taken to enter the dark compartment of the passive avoidance test during the training and test sessions (24 h and 7 days after training). Data are presented as mean (±SEM), *n* = 10–12 mice/group. *p < 0.05 and **p < 0.01, compared to all the other experimental groups. (C) Bars represent the mean (±SEM, *n* = 10–12 mice/group) of the time to reach the goal in the difficult and easy mazes in Hebb‐Williams mazes. *p < 0.05 and **p < 0.01, compared to all the other experimental groups. (D) Bars denote the mean (±SEM, *n* = 10–12 mice/group) of the number of errors to reach the goal in the difficult and easy mazes in Hebb‐Williams mazes. **p < 0.01 and ***p < 0.001, compared to their respective saline‐treated group.

In the novel object recognition test (Figure 6A), the two‐way ANOVA revealed a significant effect of the variables Ethanol [*F*(1, 43) = 4.487, *p* < 0.05], MSC‐EVs [*F*(1, 43) = 4.482, *p* < 0.05], and of the Ethanol × MSC‐EVs interaction [*F*(1, 43) = 6.006, *p* = 0.01]. The ethanol‐treated mice failed to recognize the novel object because their discrimination index was significantly lower than in the saline‐treated mice and the mice treated with MSC‐EVs plus ethanol (*p* < 0.01 in both cases).

The Kruskal‐Wallis test for the passive avoidance data (Figure 6B) showed an effect on the training day [χ^2^(2) = 12.440, *p* < 0.01] and 7 days after the training day [χ^2^(2) = 17.359, *p* < 0.001]. On the training day, the Mann–Whitney *U* test revealed longer latency to cross to the dark compartment in the animals treated with ethanol compared to all the other experimental groups (saline: *U* = 23, *p* = 0.01; ethanol plus MSC‐EVs: *U* = 17, *p* < 0.01; saline plus MSC‐EVs *U* = 10, *p* < 0.001). Conversely, 7 days after the training day, the ethanol‐treated mice crossed to the dark compartment more quickly than all other groups (saline: *U* = 10.5, *p* < 0.001; ethanol plus MSC‐EVs: *U* = 7.5, *p* < 0.001; saline plus MSC‐EVs: *U* = 23, *p* < 0.05). These findings suggest that ethanol causes poor retention in memory tasks, while MSC‐EVs administration can preserve cognition when harmful ethanol effects come into play.

The Kruskal‐Wallis test revealed an effect on the time to reach the goal in the easy and difficult mazes [χ^2^(2) = 24.500, *p* < 0.01; χ^2^(2) = 13.500, *p* = 0.001, respectively; Figure 6C]. Pairwise comparisons showed that the ethanol‐treated mice took longer to reach the goal in the easy and difficult mazes compared to the other experimental groups (saline: *U* = 24.5, *p* < 0.01; ethanol plus MSC‐EVs: *U* = 35.3, *p* < 0.05; saline plus MSC‐EVs: *U* = 27, *p* < 0.05, in the easy mazes; and saline: *U* = 13.5, *p* < 0.001; ethanol plus MSC‐EVs: *U* = 13, *p* < 0.01; saline plus MSC‐EVs: *U* = 11, *p* = 0.001, in the difficult mazes). These data (Figure 6C) and the data from Supplementary Material (Figures S2 and S3) also confirm that MSC‐EVs treatment was able to revert the impairment in the spatial learning induced by ethanol in adolescent mice.

Furthermore, the ANOVA for the total number of errors in the Hebb‐Williams maze (Figure 6D) revealed an effect of the variable Ethanol in the easy [*F*(1, 39) = 5.785, *p* < 0.05] and difficult [*F*(1, 39) = 47.493, *p* < 0.001] mazes. The animals treated with ethanol and ethanol plus MSC‐EVs made more errors while learning mazes versus the saline‐treated mice (*p* < 0.01 and *p* < 0.001 for the easy and difficult mazes, respectively; Figure 6D and Figure S4).

### 3.4 Role of MSC‐EVs in the ethanol‐induced inflammatory response in astroglial cells in primary culture

**FIGURE 7:**
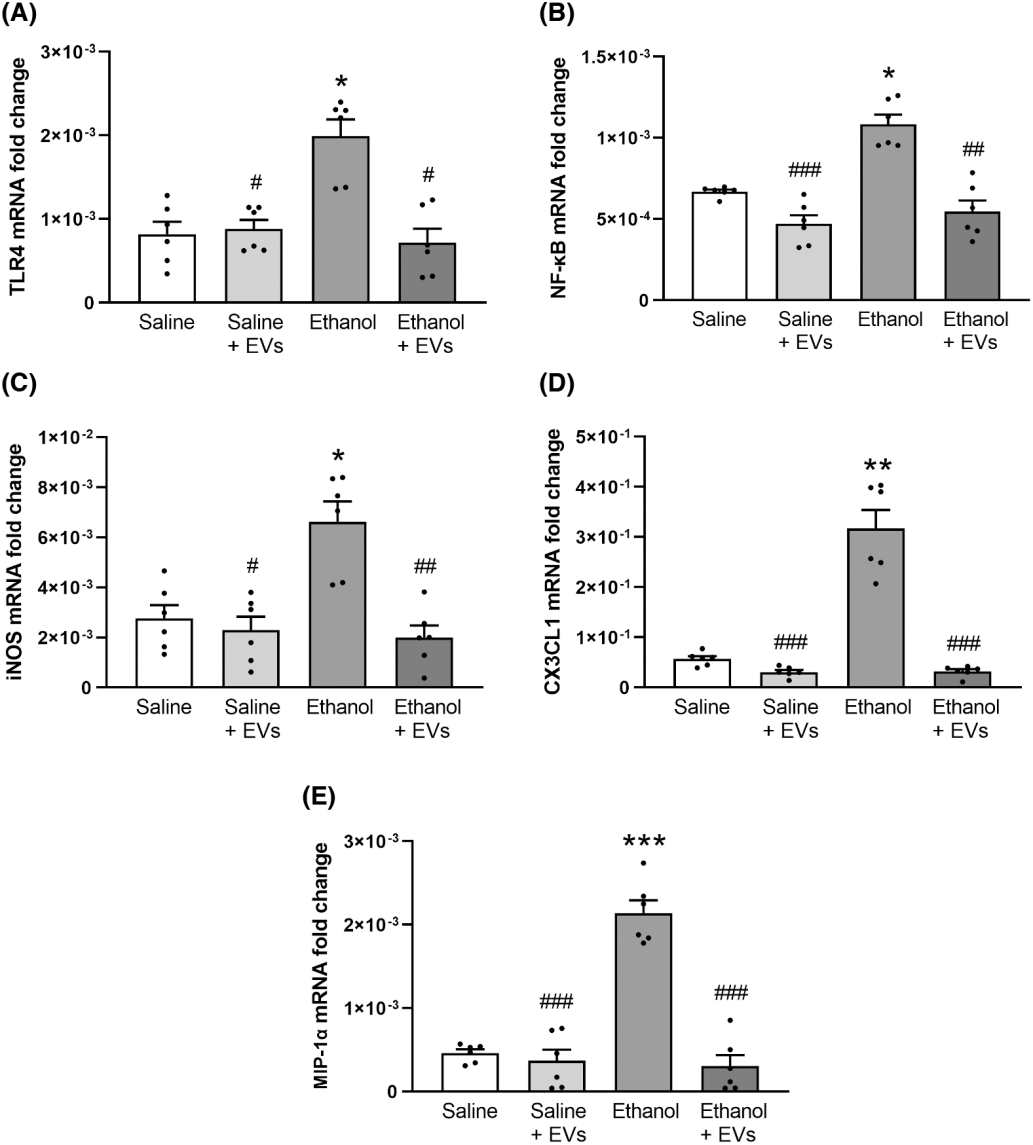
MSC‐EVs lower the levels of the inflammatory genes induced by ethanol treatment in astroglial cells. The astrocytes in primary culture treated or not with ethanol in the presence or absence of MSC‐EVs were used to analyze the mRNA levels of TLR4 (A), NF‐κB (B), iNOS (C), CX3CL1 (D) and MIP‐1α (E). Data represent mean ± SEM (*n* = 6 independent experiments). *p < 0.05, **p < 0.01 and ***p < 0.001, compared to their respective untreated cells; ^#^ *p* < 0.05, ^##^ *p* < 0.01 and ^###^ *p* < 0.001, compared to their respective ethanol‐treated cells.

We have previously demonstrated that ethanol induces neuroinflammation by activating the TLR4 inflammatory response in glial cells.^17^, ^42^ Therefore, to extend the in vivo results on the protective role of MSC‐EVs in ethanol‐induced neuroinflammation, we also used the primary culture of the astroglial cells exposed to ethanol and treated with and without MSC‐EVs. The results demonstrated that ethanol treatment increased the levels of several proinflammatory genes, such as TLR4, NF‐κB, iNOS, CX3CL1, and MIP‐1α. The one‐way ANOVA revealed that ethanol treatment increased the gene expression of TLR4 [*F* (3, 13) = 6.663, *p* < 0.05; Figure 7A], NF‐κB [*F*(3, 14) = 13.360, *p* < 0.05; Figure 7B], iNOS [*F*(3, 13) = 6.363, *p* < 0.05; Figure 7C], CX3CL1 [*F*(3, 15) = 39.440, *p* < 0.01; Figure 7D] and MIP‐1α [*F*(3, 16) = 30.990, *p* < 0.001; Figure 7E]. Notably, the treatment with MSC‐EVs significantly diminished the genes expression induced by ethanol TLR4 [*F*(3, 13) = 6.663, *p* < 0.05; Figure 7A], NF‐κB [*F*(3, 14) = 13.360, *p* < 0.01; Figure 7B], iNOS [*F*(3, 13) = 6.363, *p* < 0.01; Figure 7C], CX3CL1 [*F*(3, 15) = 39.440, *p* < 0.001; Figure 7D] and MIP‐1α [*F*(3, 16) = 30.990, *p* < 0.001; Figure 7E]. No significant changes were observed in the expression of these inflammatory genes when MSC‐EVs were added to the cell medium alone.

## 4 DISCUSSION

**FIGURE 8:**
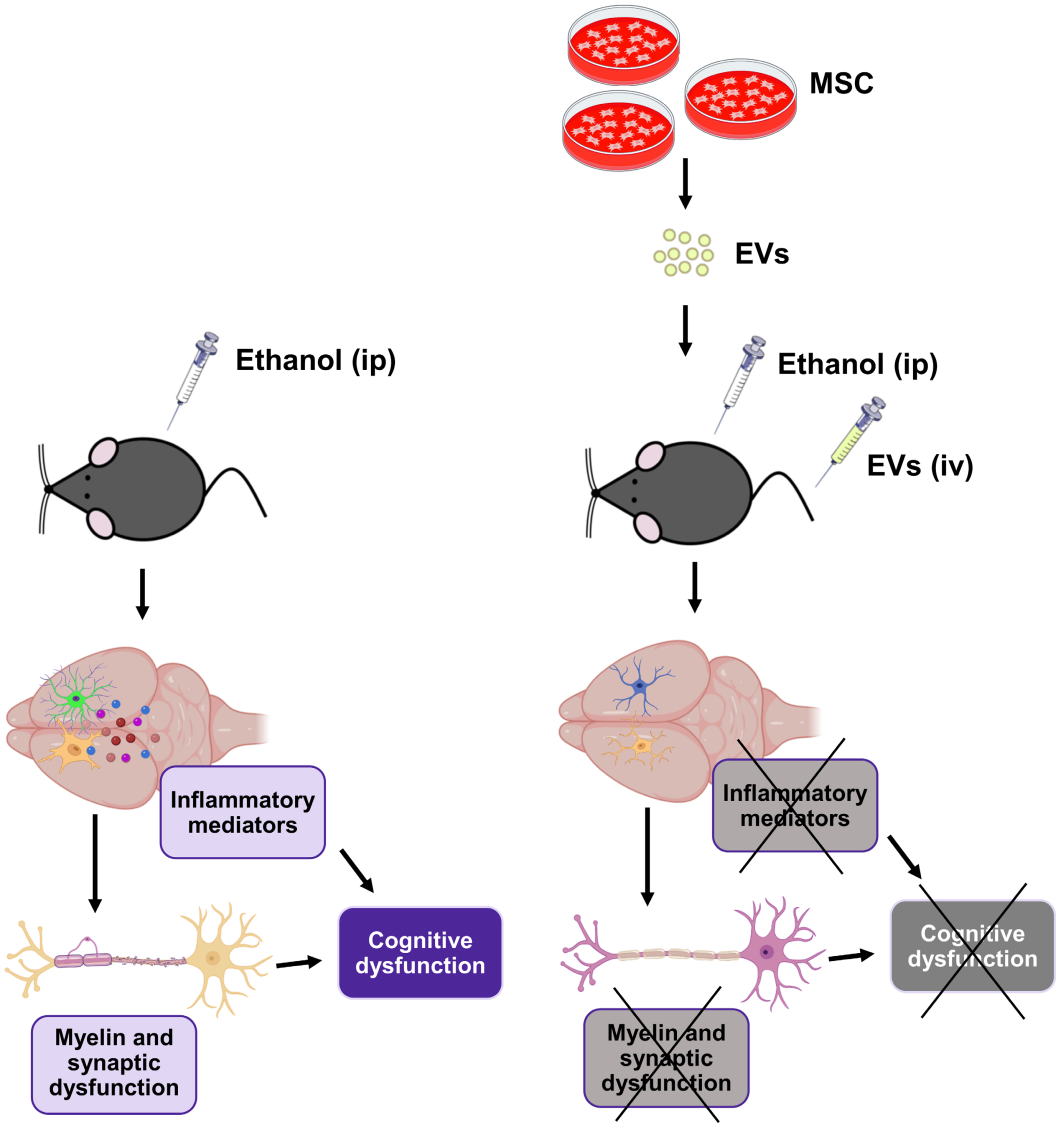
Schematic representation of the protective effects of MSC‐EVs administration on ethanol‐induced neuroinflammation. Adipose tissue‐derived EVs were administered by intravenous injection (iv) prior to ethanol treatment (ip, intraperitoneal injection) in adolescent mice. MSC‐EVs administration ameliorates ethanol‐induced up‐regulation in inflammatory genes in the prefrontal cortex of adolescent mice. Notably, MSC‐EVs treatment also restored the myelin and synaptic derangements, as well as the memory and learning impairments, induced by ethanol administration.

We have previously demonstrated the critical role of the innate immune response in the ethanol‐induced PFC damage and cognitive dysfunctions induced by binge‐like ethanol exposure in adolescence.^18^, ^25^ Considering the regenerative potential of MSC‐EVs in both brain damage and neurodegenerative disorders,^43^, ^44^, ^45^, ^46^ MSC‐EVs were herein used to ameliorate the neuroinflammation induced by binge ethanol drinking. The present findings provide evidence that MSC‐EVs administration mostly restores the neuroinflammatory response, along with myelin and synaptic structural alterations, as well as cognitive and memory dysfunctions induced by binge‐like ethanol treatment in adolescent mice (Figure 8).

Our previous studies revealed the role of EVs as amplifiers of the TLR4 response and the neuroinflammation induced by ethanol treatment in astroglial cells in culture^30^ and in in vivo brain tissue.^47^ However, different recent studies also report the potential role of EVs to show that EVs emerge as therapeutical agents of different diseases, such as neurodegenerative disorders and brain damage. Experimental studies have specifically evidenced the anti‐inflammatory and anti‐oxidative properties of MSC‐EVs in the TLR4 activation induced by its specific ligand, lipopolysaccharide (LPS). For instance, MSC‐EVs prevent LPS‐induced microglia activation and the production of inflammatory mediators in rats^44^ and microglial cells.^48^ Han et al.^49^ have shown that MSC‐EVs alleviate the brain expression of inflammatory cytokines in rats with subarachnoid hemorrhage by inhibiting the activation of nuclear transcription factor NF‐κB. In line with these findings, we show that MSC‐EVs can prevent the up‐regulation of the expression of inflammatory genes (e.g., iNOS, MIP‐1α, NF‐κB, and CX3CL1) induced by binge‐like ethanol treatment in both the adolescent PFC and ethanol‐treated astroglial cells. Similarly, Ezquer et al.^50^ have demonstrated that MSC‐EVs inhibit the neuroinflammation and glial activation induced by chronic alcohol consumption. Protective functions of these microvesicles have been shown by the miRNAs contained in MSC‐EVs, which modulate various cell signaling processes and regulate the expression of multiple target genes with anti‐oxidative and anti‐inflammatory properties.^51^ Other studies have also demonstrated that MSC‐EVs perform immunosuppressive functions by decreasing T‐ and B‐cell proliferation, by releasing immunomodulating factors (e.g., iNOS, PGE2, TSG‐6, and HLA‐G) packed in EVs.^52^ Conversely, the EVs derived by ethanol‐treated astrocytes could be internalized by neurons to increase the levels of proinflammatory molecules, by compromising their survival.^30^ However, the present study confirms that the systemic administration of MSC‐EVs exerts anti‐inflammatory properties in ethanol‐treated adolescent mice. Indeed, MSC‐EVs incorporated into glial cells and neurons could promote neuroprotection against the neuroinflammatory response and brain damage.^49^, ^53^, ^54^

Adolescence is a critical brain maturation period when neurotoxic ethanol effects can induce structural cortical alterations in humans^55^, ^56^ and experimental models,^18^ which could be associated with persistent neurobehavioral deficits, including memory and cognitive dysfunction.^18^, ^57^, ^58^ The present findings show that the intravenous MSC‐EVs injection into binge ethanol‐treated adolescent mice is able to reduce both alterations in white matter (e.g., irregular myelin fiber shapes, interlaminar splitting of myelin sheaths) and ultrastructural changes in synapses, such as a reduction in both postsynaptic thickness and synaptic vesicle number. In line with our results, a study in rhesus monkeys with cortical injury has reported that MSC‐EVs administration can promote therapeutic actions through the recovery of the structure of premotor pyramidal neurons and dendritic plasticity function.^59^ Treating brain injury with MSC‐EVs can also promote the restoration of long‐term perinatal microstructural abnormalities of white matter,^44^ and remyelination by acting directly on oligodendrocyte progenitor cells and indirectly on microglia activation.^46^ Therefore, these protective and supportive effects of MSC‐EVs can be attributed not only to their anti‐inflammatory properties,^44^ but also to the up‐regulation of myelin‐related genes^60^ and neural growth factors (i.e., BDNF, VEGF, and EGF)^61^ in brain injury.

We have previously demonstrated that alterations in myelin and synaptic structures induced by binge drinking in adolescence can lead to poor synaptic transmission efficacy, which causes cognitive impairments in adolescent mice.^18^ Recent reports show the involvement of MSC‐EVs in the protection of LPS‐induced spatio‐temporal memory deficits and learning impairments as assessed by a novel object recognition test and Barnes maze.^44^ Cognitive function restoration using MSC‐EVs has also been demonstrated in animal models of neurodegenerative diseases, such as Alzheimer's disease, brain injury, Parkinson's disease, schizophrenia, among others.^43^, ^45^, ^46^ Notably, the present study demonstrates that MSC‐EVs ameliorate ethanol‐induced spatio‐temporal memory dysfunction, as well as learning and recognition memory deficits as evaluated by the novel object recognition test, the passive avoidance task, and Hebb‐Williams maze. Indeed, we show that MSC‐EVs administration is capable of normalizing the disability to recognize the novel object, the shorter latency time during the passive avoidance test, and the longer times to complete the Hebb‐Williams mazes in adolescent mice treated with ethanol. Therefore, we hypothesize that an intravenous MSC‐EVs injection may rescue cognitive deficits by regulating inflammatory responses and structural cortical alterations. Several studies suggest that the protective role of MSC‐EVs in cognitive deficits could be attributed to the amelioration of white matter disturbances,^44^ along with the morphological changes in spine density and dendritic intersections or calcium signaling alterations.^62^ The fact that the stronger effect of MSC‐EVs on the ethanol‐treated mice was observed in Hebb‐Williams maze for the time to reach the goal, and not for the number of made errors, could be subjected to MSC‐EVs, which were unable to restore behavioral impairments by altering flexibility in strategy selection for problem‐solving.^63^ These deficits are more evident in very complex tasks, such as Hebb‐Williams mazes.^64^ Indeed Match et al.^65^ showed that adolescent rats treated with ethanol repeated the same incorrect response in succession, unlike the controls, which suggests lack of flexibility in correcting incorrect choices within a short timeframe.

Taken together, these novel results support the protective role of MSC‐EVs in not only the neuroinflammatory immune response, but also in the alterations of myelin and synaptic structures and cognitive dysfunction induced by binge‐like ethanol treatment in adolescence. These findings evidence the therapeutic potential of MSC‐EVs and may provide a new tool to treat the neuroinflammation associated with alcohol consumption and other neurodegenerative diseases.

## CONFLICT OF INTEREST STATEMENT

The authors have no conflicts of interest to declare.

## Supporting information


Data S1.

## Data Availability

The data that support the findings of this study are available on request from the corresponding author. The data are not publicly available due to privacy or ethical restrictions.

## References

[1] Fujita Y , Kadota T , Araya J , Ochiya T , Kuwano K . Clinical application of mesenchymal stem cell‐derived extracellular vesicle‐based therapeutics for inflammatory lung diseases. J Clin Med. 2018;7(10):E355. 10.3390/jcm7100355 PMC6210470 30322213

[2] Akyurekli C , Le Y , Richardson RB , Fergusson D , Tay J , Allan DS . A systematic review of preclinical studies on the therapeutic potential of mesenchymal stromal cell‐derived microvesicles. Stem Cell Rev Rep. 2015;11(1):150‐160. 25091427 10.1007/s12015-014-9545-9

[3] Janockova J , Slovinska L , Harvanova D , Spakova T , Rosocha J . New therapeutic approaches of mesenchymal stem cells‐derived exosomes. J Biomed Sci. 2021;28(1):39. 34030679 10.1186/s12929-021-00736-4 PMC8143902

[4] Hu Q , Lyon CJ , Fletcher JK , Tang W , Wan M , Hu TY . Extracellular vesicle activities regulating macrophage‐ and tissue‐mediated injury and repair responses. Acta Pharm Sin B. 2021;11(6):1493‐1512. 34221864 10.1016/j.apsb.2020.12.014 PMC8245807

[5] Varderidou‐Minasian S , Lorenowicz MJ . Mesenchymal stromal/stem cell‐derived extracellular vesicles in tissue repair: challenges and opportunities. Theranostics. 2020;10(13):5979‐5997. 32483432 10.7150/thno.40122 PMC7254996

[6] Zhu F , Chong Lee Shin OLS , Pei G , et al. Adipose‐derived mesenchymal stem cells employed exosomes to attenuate AKI‐CKD transition through tubular epithelial cell dependent Sox9 activation. Oncotarget. 2017;8(41):70707‐70726. 29050313 10.18632/oncotarget.19979 PMC5642588

[7] Pascual M , Ibáñez F , Guerri C . Exosomes as mediators of neuron‐glia communication in neuroinflammation. Neural Regen Res. 2019;15(5):796‐801. 10.4103/1673-5374.268893 PMC6990780 31719239

[8] Rashed MH , Bayraktar E , Helal GK , et al. Exosomes: from garbage bins to promising therapeutic targets. Int J Mol Sci. 2017;18(3):E538. 10.3390/ijms18030538 PMC5372554 28257101

[9] Wang J , Sun X , Zhao J , et al. Exosomes: a novel strategy for treatment and prevention of diseases. Front Pharmacol. 2017;8:300. 28659795 10.3389/fphar.2017.00300 PMC5468768

[10] Yari H , Mikhailova MV , Mardasi M , et al. Emerging role of mesenchymal stromal cells (MSCs)‐derived exosome in neurodegeneration‐associated conditions: a groundbreaking cell‐free approach. Stem Cell Res Ther. 2022;13(1):423. 35986375 10.1186/s13287-022-03122-5 PMC9389725

[11] Steinberg L . Cognitive and affective development in adolescence. Trends Cogn Sci. 2005;9(2):69‐74. 15668099 10.1016/j.tics.2004.12.005

[12] Alfonso‐Loeches S , Guerri C . Molecular and behavioral aspects of the actions of alcohol on the adult and developing brain. Crit Rev Clin Lab Sci. 2011;48(1):19‐47. 21657944 10.3109/10408363.2011.580567

[13] Casey BJ , Jones RM , Hare TA . The adolescent brain. Ann N Y Acad Sci. 2008;1124:111‐126. 18400927 10.1196/annals.1440.010 PMC2475802

[14] Guerri C , Pascual M . Impact of neuroimmune activation induced by alcohol or drug abuse on adolescent brain development. Int J Dev Neurosci. 2019;77:89‐98. 30468786 10.1016/j.ijdevneu.2018.11.006

[15] Jacobus J , Tapert SF . Neurotoxic effects of alcohol in adolescence. Annu Rev Clin Psychol. 2013;9:703‐721. doi:10.1146/annurev-clinpsy-050212-185610 23245341 PMC3873326

[16] Alfonso‐Loeches S , Pascual‐Lucas M , Blanco AM , Sanchez‐Vera I , Guerri C . Pivotal role of TLR4 receptors in alcohol‐induced neuroinflammation and brain damage. J Neurosci. 2010;30(24):8285‐8295. 20554880 10.1523/JNEUROSCI.0976-10.2010 PMC6634595

[17] Fernandez‐Lizarbe S , Pascual M , Guerri C . Critical role of TLR4 response in the activation of microglia induced by ethanol. J Immunol. 2009;183(7):4733‐4744. 19752239 10.4049/jimmunol.0803590

[18] Montesinos J , Pascual M , Pla A , et al. TLR4 elimination prevents synaptic and myelin alterations and long‐term cognitive dysfunctions in adolescent mice with intermittent ethanol treatment. Brain Behav Immun. 2015;45:233‐244. 25486089 10.1016/j.bbi.2014.11.015

[19] Friedman NP , Robbins TW . The role of prefrontal cortex in cognitive control and executive function. Neuropsychopharmacology. 2022;47(1):72‐89. 34408280 10.1038/s41386-021-01132-0 PMC8617292

[20] Tapert SF , Ozyurt SS , Myers MG , Brown SA . Neurocognitive ability in adults coping with alcohol and drug relapse temptations. Am J Drug Alcohol Abuse. 2004;30(2):445‐460. 15230085 10.1081/ada-120037387

[21] Yin K , Wang S , Zhao RC . Exosomes from mesenchymal stem/stromal cells: a new therapeutic paradigm. Biomark Res. 2019;7:8. 30992990 10.1186/s40364-019-0159-x PMC6450000

[22] Mellado‐López M , Griffeth RJ , Meseguer‐Ripolles J , Cugat R , García M , Moreno‐Manzano V . Plasma rich in growth factors induces cell proliferation, migration, differentiation, and cell survival of adipose‐derived stem cells. Stem Cells Int. 2017;2017:5946527‐5946511. 29270200 10.1155/2017/5946527 PMC5705873

[23] Muñoz‐Criado I , Meseguer‐Ripolles J , Mellado‐López M , et al. Human suprapatellar fat pad‐derived mesenchymal stem cells induce chondrogenesis and cartilage repair in a model of severe osteoarthritis. Stem Cells Int. 2017;2017:4758930‐4758912. 28769981 10.1155/2017/4758930 PMC5523339

[24] Brust V , Schindler PM , Lewejohann L . Lifetime development of behavioural phenotype in the house mouse (*Mus musculus*). Front Zool. 2015;12(Suppl 1):S17. 26816516 10.1186/1742-9994-12-S1-S17 PMC4722345

[25] Pascual M , Blanco AM , Cauli O , Miñarro J , Guerri C . Intermittent ethanol exposure induces inflammatory brain damage and causes long‐term behavioural alterations in adolescent rats. Eur J Neurosci. 2007;25(2):541‐550. 17284196 10.1111/j.1460-9568.2006.05298.x

[26] Allen‐Worthington KH , Brice AK , Marx JO , Hankenson FC . Intraperitoneal injection of ethanol for the euthanasia of laboratory mice (*Mus musculus*) and rats (*Rattus norvegicus*). J Am Assoc Lab Anim Sci. 2015;54(6):769‐778. 26632787 PMC4671793

[27] Harris JA , Mihalas S , Hirokawa KE , et al. Hierarchical organization of cortical and thalamic connectivity. Nature. 2019;575(7781):195‐202. 31666704 10.1038/s41586-019-1716-z PMC8433044

[28] Le Merre P , Ährlund‐Richter S , Carlén M . The mouse prefrontal cortex: unity in diversity. Neuron. 2021;109(12):1925‐1944. 33894133 10.1016/j.neuron.2021.03.035

[29] Pascual M , Guerri C . The peptide NAP promotes neuronal growth and differentiation through extracellular signal‐regulated protein kinase and Akt pathways, and protects neurons co‐cultured with astrocytes damaged by ethanol. J Neurochem. 2007;103(2):557‐568. 17623041 10.1111/j.1471-4159.2007.04761.x

[30] Ibáñez F , Montesinos J , Ureña‐Peralta JR , Guerri C , Pascual M . TLR4 participates in the transmission of ethanol‐induced neuroinflammation via astrocyte‐derived extracellular vesicles. J Neuroinflammation. 2019;16:136. 31272469 10.1186/s12974-019-1529-x PMC6610989

[31] Schmittgen TD , Livak KJ . Analyzing real‐time PCR data by the comparative C(T) method. Nat Protoc. 2008;3(6):1101‐1108. 18546601 10.1038/nprot.2008.73

[32] Pascual M , López‐Hidalgo R , Montagud‐Romero S , Ureña‐Peralta JR , Rodríguez‐Arias M , Guerri C . Role of mTOR‐regulated autophagy in spine pruning defects and memory impairments induced by binge‐like ethanol treatment in adolescent mice. Brain Pathol. 2021;31(1):174‐188. 32876364 10.1111/bpa.12896 PMC8018167

[33] Rabinovitch MS , Rosvold HE . A closed‐field intelligence test for rats. Can J Psychol. 1951;5(3):122‐128. 14870071 10.1037/h0083542

[34] Harris KM , Jensen FE , Tsao B . Three‐dimensional structure of dendritic spines and synapses in rat hippocampus (CA1) at postnatal day 15 and adult ages: implications for the maturation of synaptic physiology and long‐term potentiation. J Neurosci. 1992;12(7):2685‐2705. 1613552 10.1523/JNEUROSCI.12-07-02685.1992 PMC6575840

[35] Moreira PS , Almeida PR , Leite‐Almeida H , Sousa N , Costa P . Impact of chronic stress protocols in learning and memory in rodents: systematic review and meta‐analysis. PloS One. 2016;11(9):e0163245. 27662580 10.1371/journal.pone.0163245 PMC5035061

[36] Fuchsberger T , Yuste R , Martinez‐Bellver S , et al. Oral monosodium glutamate administration causes early onset of Alzheimer's disease‐like pathophysiology in APP/PS1 mice. J Alzheimers Dis. 2019;72(3):957‐975. 31658055 10.3233/JAD-190274

[37] Stanford L , Brown RE . MHC‐congenic mice (C57BL/6J and B6‐H‐2K) show differences in speed but not accuracy in learning the Hebb‐Williams maze. Behav Brain Res. 2003;144(1–2):187‐197. 12946609 10.1016/s0166-4328(03)00093-7

[38] Pascual M , Montesinos J , Montagud‐Romero S , et al. TLR4 response mediates ethanol‐induced neurodevelopment alterations in a model of fetal alcohol spectrum disorders. J Neuroinflammation. 2017;14(1):145. 28738878 10.1186/s12974-017-0918-2 PMC5525270

[39] Ródenas‐González F , Blanco‐Gandía MC , Miñarro J , Rodríguez‐Arias M . Cognitive profile of male mice exposed to a ketogenic diet. Physiol Behav. 2022;254:113883. 35716801 10.1016/j.physbeh.2022.113883

[40] Blanco‐Gandía MC , Miñarro J , Rodríguez‐Arias M . Behavioral profile of intermittent vs continuous access to a high fat diet during adolescence. Behav Brain Res. 2019;368:111891. 31009646 10.1016/j.bbr.2019.04.005

[41] Blanco‐Gandia MC , Montagud‐Romero S , Navarro‐Zaragoza J , et al. Pharmacological modulation of the behavioral effects of social defeat in memory and learning in male mice. Psychopharmacology. 2019;236(9):2797‐2810. 31049607 10.1007/s00213-019-05256-6

[42] Blanco AM , Vallés SL , Pascual M , Guerri C . Involvement of TLR4/type I IL‐1 receptor signaling in the induction of inflammatory mediators and cell death induced by ethanol in cultured astrocytes. J Immunol. 2005;175(10):6893‐6899. 16272348 10.4049/jimmunol.175.10.6893

[43] Cui GH , Guo HD , Li H , et al. RVG‐modified exosomes derived from mesenchymal stem cells rescue memory deficits by regulating inflammatory responses in a mouse model of Alzheimer's disease. Immun Ageing. 2019;16:10. 31114624 10.1186/s12979-019-0150-2 PMC6515654

[44] Drommelschmidt K , Serdar M , Bendix I , et al. Mesenchymal stem cell‐derived extracellular vesicles ameliorate inflammation‐induced preterm brain injury. Brain Behav Immun. 2017;60:220‐232. 27847282 10.1016/j.bbi.2016.11.011

[45] Harrell CR , Volarevic A , Djonov V , Volarevic V . Mesenchymal stem cell‐derived exosomes as new remedy for the treatment of neurocognitive disorders. Int J Mol Sci. 2021;22(3):1433. 33535376 10.3390/ijms22031433 PMC7867043

[46] Zhang J , Buller BA , Zhang ZG , et al. Exosomes derived from bone marrow mesenchymal stromal cells promote remyelination and reduce neuroinflammation in the demyelinating central nervous system. Exp Neurol. 2022;347:113895. 34653510 10.1016/j.expneurol.2021.113895 PMC12050987

[47] Ibáñez F , Montesinos J , Area‐Gomez E , Guerri C , Pascual M . Ethanol induces extracellular vesicle secretion by altering lipid metabolism through the mitochondria‐associated ER membranes and sphingomyelinases. Int J Mol Sci. 2021;22(16):8438. 34445139 10.3390/ijms22168438 PMC8395151

[48] Jaimes Y , Naaldijk Y , Wenk K , Leovsky C , Emmrich F . Mesenchymal stem cell‐derived microvesicles modulate lipopolysaccharides‐induced inflammatory responses to microglia cells. Stem Cells. 2017;35(3):812‐823. 27862694 10.1002/stem.2541

[49] Han M , Cao Y , Guo X , et al. Mesenchymal stem cell‐derived extracellular vesicles promote microglial M2 polarization after subarachnoid hemorrhage in rats and involve the AMPK/NF‐κB signaling pathway. Biomed Pharmacother. 2021;133:111048. 33378955 10.1016/j.biopha.2020.111048

[50] Ezquer F , Quintanilla ME , Morales P , et al. Intranasal delivery of mesenchymal stem cell‐derived exosomes reduces oxidative stress and markedly inhibits ethanol consumption and post‐deprivation relapse drinking. Addict Biol. 2019;24(5):994‐1007. 30239077 10.1111/adb.12675

[51] Luo Q , Xian P , Wang T , et al. Antioxidant activity of mesenchymal stem cell‐derived extracellular vesicles restores hippocampal neurons following seizure damage. Theranostics. 2021;11(12):5986‐6005. 33897894 10.7150/thno.58632 PMC8058724

[52] Chen SY , Lin MC , Tsai JS , et al. EP4 antagonist‐elicited extracellular vesicles from mesenchymal stem cells rescue cognition/learning deficiencies by restoring brain cellular functions. Stem Cells Transl Med. 2019;8(7):707‐723. 30891948 10.1002/sctm.18-0284 PMC6591556

[53] Go V , Bowley BGE , Pessina MA , et al. Extracellular vesicles from mesenchymal stem cells reduce microglial‐mediated neuroinflammation after cortical injury in aged rhesus monkeys. GeroScience. 2020;42(1):1‐17. 31691891 10.1007/s11357-019-00115-w PMC7031476

[54] Xin D , Li T , Chu X , et al. Mesenchymal stromal cell‐derived extracellular vesicles modulate microglia/macrophage polarization and protect the brain against hypoxia‐ischemic injury in neonatal mice by targeting delivery of miR‐21a‐5p. Acta Biomater. 2020;113:597‐613. 32619670 10.1016/j.actbio.2020.06.037

[55] Bava S , Frank LR , McQueeny T , Schweinsburg BC , Schweinsburg AD , Tapert SF . Altered white matter microstructure in adolescent substance users. Psychiatry Res. 2009;173(3):228‐237. 19699064 10.1016/j.pscychresns.2009.04.005 PMC2734872

[56] Infante MA , Eberson SC , Zhang Y , et al. Adolescent binge drinking is associated with accelerated decline of gray matter volume. Cereb Cortex. 2022;32(12):2611‐2620. 34729592 10.1093/cercor/bhab368 PMC9201595

[57] Nguyen‐Louie TT , Matt GE , Jacobus J , et al. Earlier alcohol use onset predicts poorer neuropsychological functioning in young adults. Alcohol Clin Exp Res. 2017;41(12):2082‐2092. 29083495 10.1111/acer.13503 PMC5711576

[58] Squeglia LM , Schweinsburg AD , Pulido C , Tapert SF . Adolescent binge drinking linked to abnormal spatial working memory brain activation: differential gender effects. Alcohol Clin Exp Res. 2011;35(10):1831‐1841. 21762178 10.1111/j.1530-0277.2011.01527.x PMC3183294

[59] Medalla M , Chang W , Calderazzo SM , et al. Treatment with mesenchymal‐derived extracellular vesicles reduces injury‐related pathology in pyramidal neurons of monkey perilesional ventral premotor cortex. J Neurosci. 2020;40(17):3385‐3407. 32241837 10.1523/JNEUROSCI.2226-19.2020 PMC7178914

[60] Go V , Sarikaya D , Zhou Y , et al. Extracellular vesicles derived from bone marrow mesenchymal stem cells enhance myelin maintenance after cortical injury in aged rhesus monkeys. Exp Neurol. 2021;337:113540. 33264634 10.1016/j.expneurol.2020.113540 PMC7946396

[61] Kaminski N , Köster C , Mouloud Y , et al. Mesenchymal stromal cell‐derived extracellular vesicles reduce neuroinflammation, promote neural cell proliferation and improve oligodendrocyte maturation in neonatal hypoxic‐ischemic brain injury. Front Cell Neurosci. 2020;14:601176. 33362471 10.3389/fncel.2020.601176 PMC7758466

[62] Wang H , Liu Y , Li J , et al. Tail‐vein injection of MSC‐derived small extracellular vesicles facilitates the restoration of hippocampal neuronal morphology and function in APP/PS1 mice. Cell Death Discov. 2021;7(1):230. 34482379 10.1038/s41420-021-00620-y PMC8418600

[63] Sey NYA , Gómez‐A A , Madayag AC , Boettiger CA , Robinson DL . Adolescent intermittent ethanol impairs behavioral flexibility in a rat foraging task in adulthood. Behav Brain Res. 2019;373:112085. 31319133 10.1016/j.bbr.2019.112085 PMC6682461

[64] Miller KM , Risher ML , Acheson SK , et al. Behavioral inefficiency on a risky decision‐making task in adulthood after adolescent intermittent ethanol exposure in rats. Sci Rep. 2017;7(1):4680. 28680108 10.1038/s41598-017-04704-7 PMC5498633

[65] Macht V , Elchert N , Crews F . Adolescent alcohol exposure produces protracted cognitive‐behavioral impairments in adult male and female rats. Brain Sci. 2020;10(11):785. 33126417 10.3390/brainsci10110785 PMC7692738

