# Supplementary material for: Therapeutic role of mesenchymal stem cell‐derived extracellular vesicles in neuroinflammation and cognitive dysfunctions induced by binge‐like ethanol treatment in adolescent mice
Source: CNS Neurosci Ther. 2023 Jun 28;29(12):4018–31. doi: 10.1111/cns.14326 (PMC10651955; doi:10.1111/cns.14326)
Supplement: Supplementary file 1 — Data S1. [file CNS-29-4018-s001.docx]

**SUPPLEMENTARY MATERIAL**

**Table S1.** Nucleotide sequences of the primers used for the RT-PCR of genes.

| **Gene** | **Accession Number** | **Primer sequences (5’ to 3’)** |
| --- | --- | --- |
| **COX-2** | NM_011198.5 | F: CATTGACCAGAGCAGAGAGATG  R: GGCTTCCAGTATTGAGGAGAAC |
| **iNOS** | NM_001313921.1 | F: AATCTTGGAGCGAGTTGTGG  R: ATCTCTGCCTATCCGTCTCG |
| **MIP-1α** | NM_011337.2 | F: AGATTCCACGCCAATTCATC  R: CTCAAGCCCCTGCTCTACAC |
| **NF-κB** | NM_001410442.1 | F: TACCCTCAGAGGCCAGAAGA  R: CAGTTCCGTAGGGATCATCG |
| **CX3CL1** | NM_009142.3 | F: TGCGAAATCATGTGCGACAA  R: TGGACCCATTTCTCCTTCGG |
| **MCP-1** | NM_011333.3 | F: AGGTCCCTGTCATGCTTCTG  R: TCTGGACCCATTCCTTCTTG |
| **TLR4** | NM_021297.3 | F: TGCCTCTCTTGCATCTGGCTGG  R: CTGTCAGTACCAAGGTTGAGAGCTGG |
| **MBP** | NM_021297.3 | F: GGAGCCCTCTGCCCTCTCATGCCC  R: CCCTCGGCCCCCTTCCCTTGGGA |
| **MAG** | NM_021297.3 | F: GGCTGGGCCACGAGGGGCTGGGAG  R: GCAGCGGCGGCGGGTTGCTGTC |
| **CNPase** | NM_021297.3 | F: GCGGCCCCGGAGACATAGTA  R: TGAGCCGAGCCAGTGTGGACT |
| **NG2** | NM_021297.3 | F: TTCCCGAGGGGCGGCACCCGGACG  R: CTGCAGGGCCCCGTGCTGCGGT |
| **Cyclophilin A** | NM_008907.2 | F: GTCTCCTTCGAGCTGTTTGC  R: GATGCCAGGACCTGTATGCT |

**FIGURES**


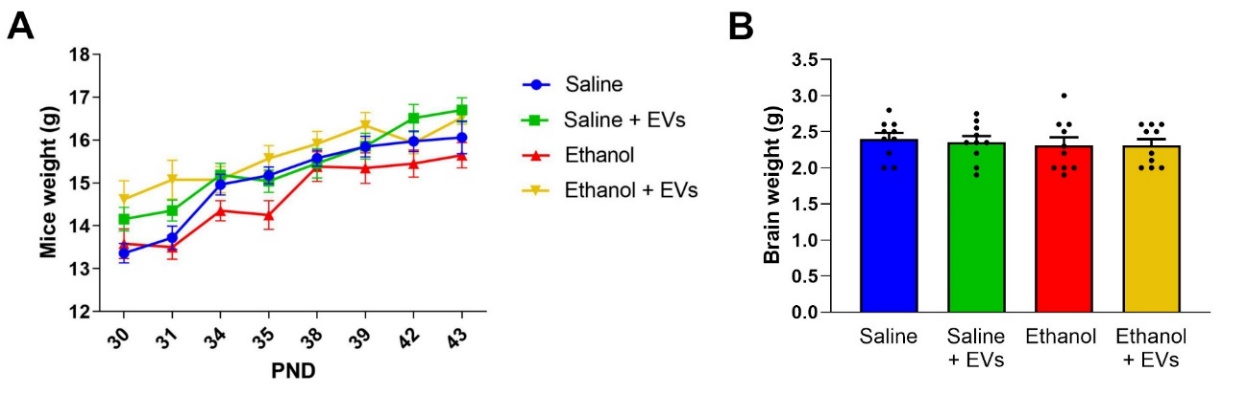
**Figure S1.** Body weights in the mice treated intermittently with saline, saline plus EVs, ethanol and ethanol plus EVs for 2 weeks (PND30 to PND43). **(A)** Body weights were measured prior to the intraperitoneal injection on the indicated postnatal day (PND). **(B)** Brain weights were quantified after sacrificing animals on PND44. Values represent mean±SEM, n=10 mice/group.


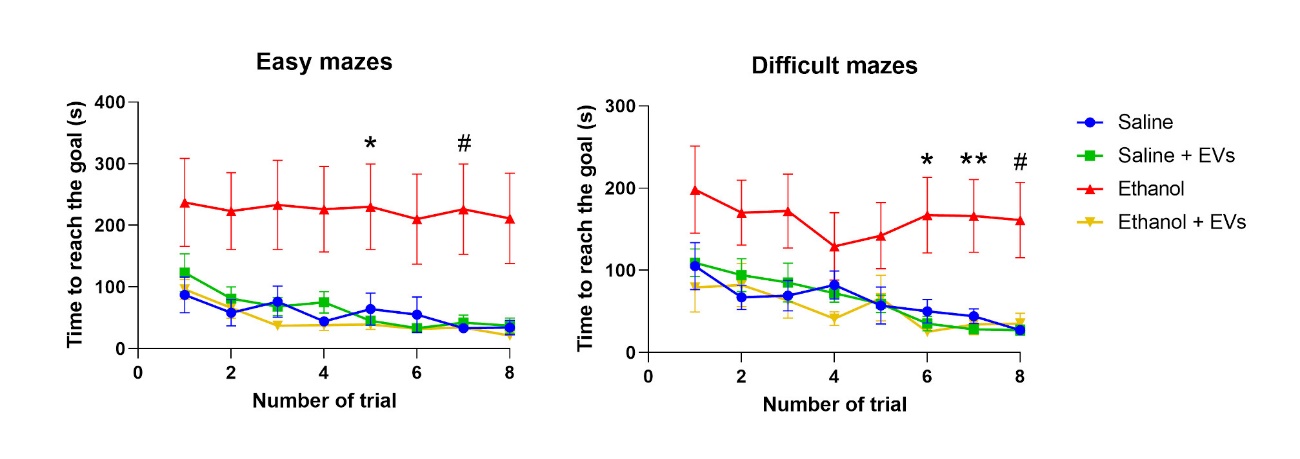
**Figure S2.** Graphs represent the mean (±SEM, n=12 mice/group) of the latency to reach the goal in each trial in the difficult and easy mazes in Hebb-Williams mazes. * p < 0.05 and ** p < 0.01, the ethanol-treated mice compared to ethanol plus MSC-EVs- and saline plus MSC-EVs-treated mice. # p < 0.05, the ethanol-treated mice compared to the other groups.

The Kruskal-Wallis test of the latency to reach the goal in each trial, of the difficult and easy mazes, revealed an effect of latency to reach the goal in each trial in the easy and difficult mazes for the 5^th^ (χ2(2) = 7.554, p = 0.05), and 7^th^ (χ2(2) = 8.289, p < 0.05) trial of the easy mazes; and for the 6^th^ (χ2(2) = 11.095, p < 0.05), 7^th^ (χ2(2) = 9.786, p < 0.05) and 8^th^ (χ2(2) = 7.978, p < 0.05) trials for the difficult mazes. In the easy mazes, the Mann-Whitney U test showed that the ethanol-treated mice took longer to reach the goal in the 5^th^ trial compared to the saline (U = 33.500, p < 0.05) and ethanol plus MSC-EVs groups (U = 24.500, p < 0.05), and also in the 7^th^ trial compared to all the other experimental groups (saline: U = 25.500, p = 0.01; ethanol plus MSC-EVs: U = 29.500, p < 0.05; saline plus MSC-EVs: U = 27.500, p < 0.05).

In the difficult mazes the pairwise comparisons showed that the ethanol-treated mice took longer to reach the goal in the 6^th^ trial compared to the animals treated with ethanol plus MSC-EVs (U = 13.500, p<0.01) and saline plus MSC-EVs (U = 23.000, p < 0.05), in the 7^th^ trial compared to the animals treated with ethanol plus MSC-EVs (U = 20.500, p<0.01) and saline plus MSC-EVs (U = 21.000, p < 0.01), and in the 8^th^ trial compared to all the other experimental groups (saline: U = 30.500, p < 0.05; ethanol plus MSC-EVs: U = 26.000, p<0.01; saline plus MSC-EVs: U = 25.500, p < 0.05).

**Figure S3.** Graphs represent the mean (±SEM, n=12 mice/group) of the latency to reach the goal in the different Hebb-Williams mazes. * p < 0.05 and ** p < 0.01, compared to their respective saline-treated group. # p < 0.05 and ## p < 0.01, compared to their respective ethanol-treated group. ++p < 0.01, compared to their respective saline plus MSC-EVs-treated group.

Easy mazes:


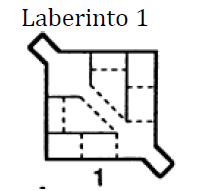


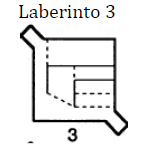

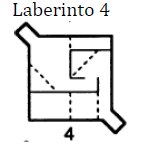

Difficult mazes:


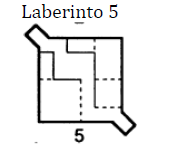

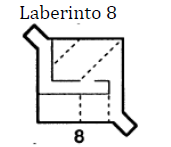


Shapiro–Wilk test was used to test data for normality distribution in the Hebb-Williams test for the 1^st^, 3^rd^, 4^th^, 5^th^ and 8^th^ mazes. Then, Kruskal-Wallis tests and the Mann-Whitney U test for the pairwise comparisons were used.

The time to reach the goal in the different mazes of the Hebb-Williams test were analyzed with the Kruskal-Wallis test. The results showed an effect of the treatment in the 1^st^ (χ2(2) = 7.945, p < 0.05), 3^rd^ (χ2(2) = 14.175, p < 0.01), 5^th^ (χ2(2) = 10.102, p < 0.05), and 8^th^ (χ2(2) = 17.729; p < 0.001) mazes. The ethanol-treated mice took longer to reach the goal in these four mazes (1^st^ maze: U = 30.500, p < 0.05; 3^rd^ maze: U = 31.000, p < 0.05; 5^th^ maze: U = 20.000, p < 0.01; 8^th^ maze: U = 17.729; p < 0.05) when compared to the saline-treated mice. In addition, the ethanol-treated mice took longer to reach the goal, compared to the saline plus MSC-EVs-treated mice in the 5^th^ maze (U = 14.000, p < 0.01) and ethanol plus MSC-EVs-treated mice in the 1^st^ (U = 30.000, p < 0.05) and 8^th^ (U = 13.000, p < 0.01) mazes.

**Figure S4.** Graphs represent the mean (±SEM, n=12 mice/group) of the number of errors to reach the goal in the difficult and easy mazes in Hebb-Williams mazes. * p < 0.05 and ** p < 0.01, the ethanol-treated mice compared to the saline- and saline plus MSC-EVs-treated mice. ## p < 0.01, the ethanol-treated mice compared to the saline plus MSC-EVs-treated mice.


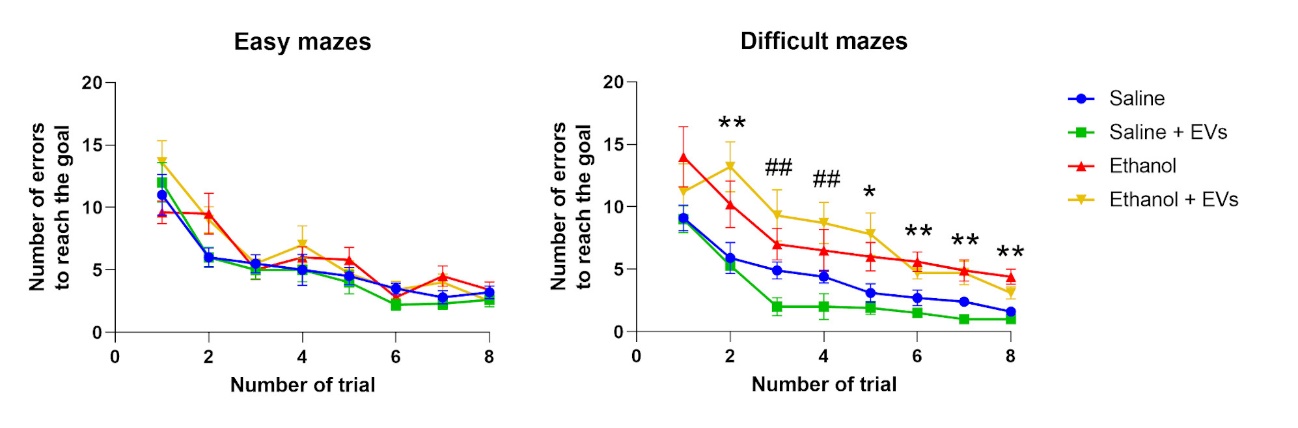


The Kruskal-Wallis test for the number of errors to reach the goal in each trial revealed a significant effect only on the difficult mazes for the 2^nd^ (χ2(2) = 21.549, p < 0.001), 3^rd^ (χ2(2) = 17.725, p < 0.001), 4^th^ (χ2(2) = 18.646, p < 0.001), 5^th^ (χ2(2) = 15.580, p < 0.001), 6^th^ (χ2(2) = 20.659, p < 0.001), 7^th^ (χ2(2) = 26.150, p < 0.001) and 8^th^ (χ2(2) = 29.770, p < 0.001) trials. Pairwise comparisons showed that the ethanol-treated mice made more errors to reach the goal in the 2^nd^ trial (compared to the saline U = 24.500, p < 0.01 and saline plus MSC-EVs U = 14.000, p < 0.001); in the 3^rd^ and 4^th^ trials (compared to saline plus MSC-EVs U = 12.000, p < 0.001 and U = 27.000, p < 0.01, respectively); in the 5^th^ (compared to saline U = 39.000, p < 0.05 and saline plus MSC-EVs U = 22.500, p < 0.01); 6^th^ (compared to saline U = 28.500, p = 0.01 and saline plus MSC-EVs U = 16.000, p < 0.001); 7^th^ (compared to saline U = 29.500, p = 0.01 and saline plus MSC-EVs U = 2.500, p < 0.001); 8^th^ (compared to saline U = 15.000, p < 0.01 and saline plus MSC-EVs U = 3.000, p < 0.001).
